# Genome-wide pharmacologic unmasking identifies tumor suppressive microRNAs in multiple myeloma

**DOI:** 10.18632/oncotarget.4769

**Published:** 2015-07-03

**Authors:** Chonglei Bi, Tae-Hoon Chung, Gaofeng Huang, Jianbiao Zhou, Junli Yan, Gregory J. Ahmann, Rafael Fonseca, Wee Joo Chng

**Affiliations:** ^1^ Experimental Therapeutics, Cancer Science Institute of Singapore, Singapore; ^2^ Department of Medicine, Yong Loo Lin School of Medicine, National University of Singapore, Singapore; ^3^ Mayo Clinic, Scottsdale, Arizona, USA; ^4^ Department of Hematology-Oncology, National University Cancer Institute, National University Health System, Singapore

**Keywords:** tumor suppressor, epigenetics, microRNA, myeloma

## Abstract

Epigenetic alterations have emerged as an important cause of microRNA (miRNA) deregulation. In Multiple Myeloma (MM), a few tumor suppressive miRNAs silenced by DNA hypermethylation have been reported, but so far there are few systemic investigations on epigenetically silenced miRNAs. We conducted genome-wide screening for tumor suppressive miRNAs epigenetically silenced in MM. Four Human MM Cell lines were treated with demethylating agent 5′azacytidine (5′aza). Consistently upregulated miRNAs include miR-155, miR-198, miR-135a*, miR-200c, miR-125a-3p, miR-188-5p, miR-483-5p, miR-663, and miR-630. Methylation array analysis revealed increased methylation at or near miRNA-associated CpG islands in MM patients. Ectopic restoration of miR-155, miR-198, miR-135a*, miR-200c, miR-663 and miR-483-5p significantly repressed MM cell proliferation, migration and colony formation. Furthermore, we derived a 33-gene signature from predicted miRNA target genes that were also upregulated in MM patients and associated with patient survival in three independent myeloma datasets. In summary, we have revealed important, epigenetically silenced tumor suppressive miRNAs by pharmacologic reversal of epigenetic silencing.

## INTRODUCTION

Multiple myeloma (MM) is a plasma cell (PC) malignancy characterized by clonal accumulation of plasma cells in the bone marrow.[1] MM usually develops from an asymptomatic premalignant stage called monoclonal gammopathy of undetermined significance (MGUS). This benign condition can progress to myeloma or related malignancies at a rate of ~1% per year.[2] In a fraction of patients, tumor could also occur in extramedullary sites such as blood, a condition called plasma cell leukemia (PCL).[3] Despite considerable improvements of patient survival and treatment options, it remains largely incurable and novel treatment strategies are needed.[4]

MiRNAs are ~20-nucleotide, single strand, genome-encoded RNAs with a primary role in post-transcriptional silencing through imperfect pairing with the 3′ Untranslated Region (3′UTR) of protein-coding transcripts in animals. They are highly conserved across different species and regulate most cellular processes. Deregulation of miRNA is implicated in pathogenesis and malignant progression of solid and hematological malignancies.[5] Reduced miRNA expression is often observed, suggesting that some of these miRNAs act as tumor suppressors (TS).[6] The silencing of TS-miRNAs leads to activation of oncogenes and contribute to carcinogenesis.[7] Although several mechanisms contributing to abnormal silencing of miRNAs have been discovered, repression of TS-miRNA by epigenetic mechanism such as promoter hypermethylation and/or inhibitory histone modification has emerged as a major cause.[8, 9] A number of TS-miRNAs silenced in MM have been identified, such as the miR-194-2-192 cluster, miR-34b/c and miR-203 whose promoters are frequently hypermethylated.[10-12]

In this study, we conducted genome-wide screening for TS-miRNAs in MM using 5′azacytidine (5′aza) which induces DNA hypomethylation and gene reactivation by inhibiting the action of DNA Methyltransferases (DNMTs).[13] We identified miRNAs that were consistently upregulated by 5′aza, including miRNAs previously reported to be downregulated in MM patients for unknown reason, and miRNAs with known tumor suppressor functions in other cancers but had not been reported in MM. These miRNAs showed increased level of methylation in MM patients compared with normal plasma cells. Synthetic mimics of these miRNAs reduced MM cell proliferation, migration and colony formation. In addition, these miRNAs were predicted to target mRNAs upregulated in MM including a gene signature relevant to patient survival.

## RESULTS

### Specific miRNAs are consistently upregulated by 5′aza

Four MM cell lines (H929, MM1S, OPM2, 8226) were treated with 5′aza or DMSO control, and the expression profiles of miRNAs were generated by miRNA expression profiling. 5′aza produced widespread and consistent change in miRNA expression. 74 miRNAs were upregulated by 1.5 fold or more, in at least 2 cell lines. These miRNAs were scattered on all chromosomes except chr17 and Y chromosome, with a few instances of multiple miRNAs in close proximity being simultaneously upregulated. (Supplementary Table S1) Among the miRNAs commonly upregulated by 5′aza, miR-125a-3p, miR-135a*, miR-188-5p, miR-198, miR-200c and miR-155 were reported previously to be under-expressed in MM as compared with normal plasma cells.[14, 15] MiR-135a*, miR-188-5p, miR-663 and miR-483-5p were downregulated in plasma cell leukemia.[16] Moreover, miR-483-5p, miR-663 and miR-630 had been reported to have tumor suppressor properties in other cancers.[17-19] These 9 miRNAs were selected as leading TS-miRNA candidates. (Table 1) The result of miRNA array was validated using TaqMan miRNA assay (Supplementary Figure S1). To validate that the upregulation of miRNA was due to demethylation, Methylation-Specific PCR was conducted on representative miRNAs with clearly defined CpG islands near the transcription start site (TSS) of the miRNA or its host gene. Indeed, 5′aza treatment reduced the ratio of overall methylated molecules to unmethylated molecules (Supplementary Figure S2).

**Table 1:** Expression change of selected candidate TS-miRNAs after 5′aza treatment in four human myeloma cell lines

	H929		MM1S		OPM2		8226	
systematic name	Fold Change	Regulation	Fold Change	Regulation	Fold Change	Regulation	Fold Change	Regulation
hsa-miR-125a-3p	35.19	up	126.42	up	54.71	up	102.25	up
hsa-miR-135a*	55.11	up	5.36	up	10.97	up	37.37	up
hsa-miR-188-5p	2.67	up	618.7	up	2.84	up	6.17	up
hsa-miR-155	8.52	up	2.6	up	n.d.		n.d.	
hsa-miR-198	n.d.		13.22	up	n.d.		14.41	up
hsa-miR-200c	n.d.		36.26	up	n.d.		55.71	up
hsa-miR-483-5p	2.6	up	297.01	up	131.91	up	343.55	up
hsa-miR-663	107.25	up	445.96	up	2.01	up	2.9	up
hsa-miR-630	2.63	up	50.31	up	2.72	up	7.14	up

### miRNAs consistently upregulated by 5′aza are frequently hypermethylated in MM patients

**Figure 1:**
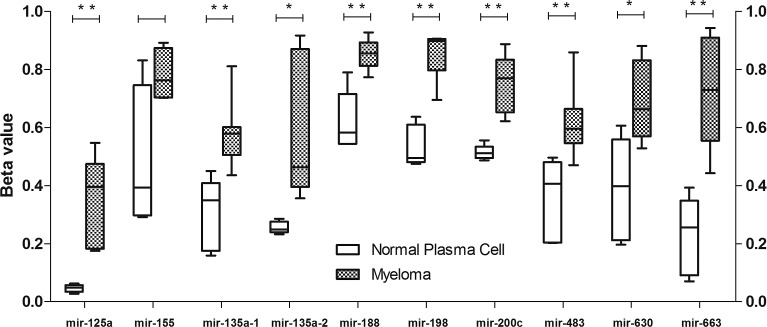
Methylation analysis of miRNA-associated CpG sites in myeloma patients and normal plasma cells Methylation data was presented as beta values, with 0 indicating 0 % DNA methylation and 1 indicating 100 % DNA methylation. The data was presented using box plot where the upper and lower whiskers denoted maximum and minimum data respectively. Student's t-test was used for p-value calculation. Asterisk (*) denotes p<0.05 and (**) denotes p<0.01.

Hypermethylation of CpG sites within the promoters (often marked by presence of CpG islands) of genes is often associated with suppression of transcription, a feature often found in human cancers.[20] Recent studies have shown that not only in promoters, the hypermethylation of CpG sites in CpG island shores as well as intragenic and intergenic CpG sites can also lead to silencing.[21-23] Therefore, the methylation status of CpG sites near the TSS of pre-miRNAs, both upstream and downstream, and host genes of the 9 TS-miRNA candidates was examined in 17 clincal MM samples and 5 normal controls profiled using Illumina methylation 450 array. Consistent increase in methylation level was observed in all 9 TS-miRNA candidates, as compared with healthy controls (Figure 1 and Supplementary Table S2).

### *In silico* functional analysis of predicted targets of candidate TS-miRNAs

To explore the mechanism of action for these candidate TS-miRNAs, we next analyzed the predicted mRNA targets of each miRNA. To reduce false positive predictions, only those genes predicted by at least half of available algorithms were selected for further analysis. 1354 unique genes were predicted to be the targets of the 9 leading candidate TS-miRNAs. Among these targets, 215 were upregulated in MM compared with normal PCs in a large public Myeloma Gene Expression Profile dataset [24] (Myeloma Institute for Research and Therapy, University of Arkansas for Medical Sciences, GEO accession number GSE2658; using a cutoff of 1.5 fold and corrected p-value of 0.005). These 215 genes (Supplementary Table S3) included oncogenes such as CCND1, BCL9, and WHSC1. The details of their upregulation in MM and their functional relevance in cancer were listed in Supplementary Table S4.

Gene Set Enrichment Analysis (GSEA) [25] of Molecular Signatures Database V3.0 showed significant enrichment of four gene sets in MM-normal phenotype comparison using the 215-gene list, including “genes downregulated in MM cell lines treated with demethylating agent decitabine and HDAC inhibitor TSA”,[26] which was consistent with miRNA target prediction since the downregulation could be attributed to the re-expression of miRNAs upon treatment, which in turn repressed the expression of their targets (Supplementary Table S5).

KEGG pathway analysis[27] revealed significant enrichment of the 215 predicted targets in Adherens junction pathway (p=0.032) among others (Supplementary Table S6), suggesting involvement of these genes in cell adhesion and migration.

### Restoration of miRNA expression reduced cell viability, migration and colony formation

**Figure 2:**
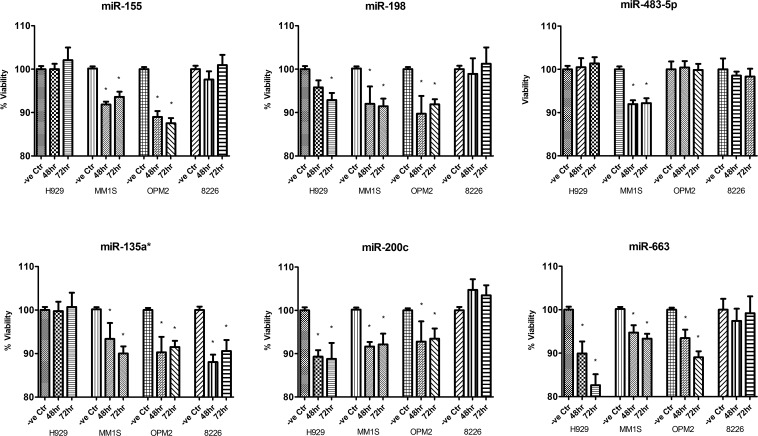
CellTiter-Glo assay measuring cellular viability of MM cells after overexpression of miRNA mimics compared with MM cells transfected with non-targeting miRNA mimics Each mimic was transfected three times in independent experiments and each sample was assayed in triplicate. The data were presented as mean +/− SEM. –ve Ctr: non-targeting miRNA mimic control; Asterisk (*) denotes statistically significance p<0.05.

To directly assess the functional relevance of these miRNAs, synthetic mimic of these miRNAs were transfected into MM cells. Their re-expression was confirmed by TaqMan miRNA assay. CellTiter-Glo assay revealed significant, 10-20% decrease in number of viable cells 48 and 72 hours after transfection, compared with negative control (non-targeting sequence based on *C.elegans* miRNAs) (Figure 2).

**Figure 3:**
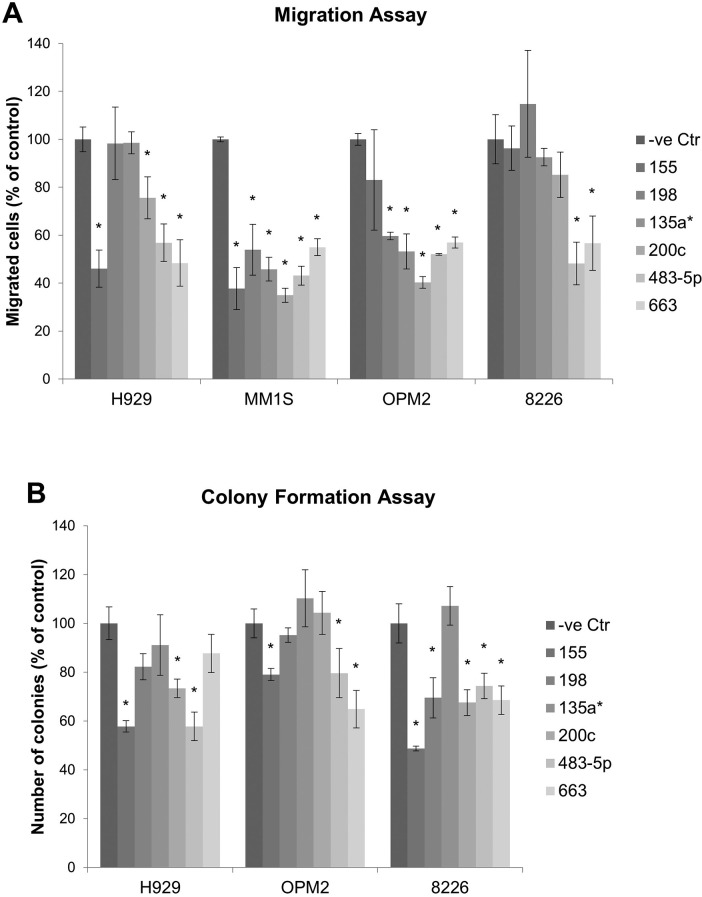
Migration and colony formation assay measuring the effect of miRNA mimic on migration and colony formation of MM cells (**A**). Migration assay comparing the migratory ability of MM cells transfected with miRNA to negative control. (**B**). Colony formation assay. MM1S cells formed very few colonies and were excluded for the assay. Each experiment was performed in triplicate. The data were presented as mean +/− SEM. Asterisk (*) denotes statistical significance p<0.05.

Migration assay was conducted to compare the effect on migration of the miRNAs. The result showed significant repression of MM cell migration by miR-155, miR-198, miR-135a*, miR-200c, miR-483-5p and miR-663 (Figure 3A), Furthermore, the effect of miRNA on clonogenicity was examined using colony formation assay. MiR-155, miR-198, miR-200c, miR-483-5p and miR-663 significantly suppressed colony formation in MM cells (Figure 3B). The result of functional study was summarized in Table 2.

**Table 2:** Summary of functional study of selected miRNA candidates

	Viability				Migration				Colony Formation		
	H929	MM1S	OPM2	8226	H929	MM1S	OPM2	8226	H929	OPM2	8226
miR-155		+	+		+	+			+	+	+
miR-198	+	+	+			+	+				+
miR-483-5p		+			+	+	+	+	+	+	+
miR-135a*		+	+	+		+	+				
miR-200c	+	+	+		+	+	+		+		+
miR-663	+	+	+		+	+	+	+		+	+

“+” **denotes observed suppression of viability/migration/colony formation.** MM1S did not form any colonies.

### Gene expression signature of TS-miRNA target genes is associated with patient survival

**Figure 4:**
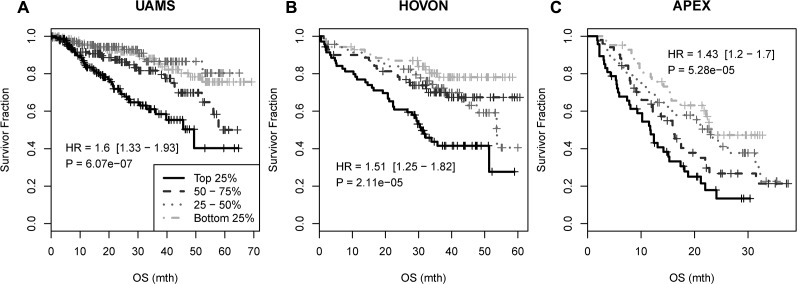
Survival association of the 33-gene signature in UAMS, HOVON and APEX myeloma datasets The survival association was tested by grouping RS values of a dataset in 4 equally-spaced levels: OS: overall survival.

To assess the clinical relevance of the predicted miRNA targets, the association between survival and expression of the 215 genes was tested in UAMS dataset (GSE2658). Of 33 genes independently associated with survival (p<0.05, Cox regression, Supplementary Table S7), 19 showed positive association such that higher expression made patient riskier (C^+^ group) whereas 14 showed negative association (C^−^ group). The survival signature was constructed using all 33 genes. The risk score (RS) for each sample was calculated as RS = U – D where U = median (E_C+_) and D = median (E_C−_) where E_C+_ is the median expression level of C^+^ group probesets/genes and E_C−_ is the median expression level of C^−^ group probesets/genes. The survival association was tested by grouping RS values of a dataset in 4 equally-spaced levels. As expected, the signature was strongly associated with overall survival in the UAMS dataset (Figure 4A). Two other independent myeloma datasets HOVON (GSE19784) and APEX (GSE9782) were used to validate the prognostic significance of the signature.[28, 29] Again it was strongly associated with survival with Hazard Ratio (HR) of 1.51, 95% Confidence Interval (CI) of [1.25-1.82] and p-value of 2.11 × 10^−5^ in HOVON dataset, and HR of 1.43, 95% CI [1.2-1.7] and p-value of 5.28 × 10^−5^ in the APEX dataset (Figure 4B and 4C).

## DISCUSSION

Several studies have shown that miRNAs are critical players in MM. A number of oncogenic and TS-miRNAs have been identified. For example, miRNA-17 ~ 92 cluster is overexpressed in MM patients and has oncogenic properties [14], whereas the silencing of tumor suppressor miR-194-2-192 miRNA cluster impairs the p53/MDM2 pathway and favors MM development. Epigenetic silencing, primarily DNA hypermethylation, has been established as a major cause for the repression of at least half of the TS-miRNA identified so far in MM, including miR-34 family, miR-194-2-192 cluster, and miR-203. In this study, we adopted a systemic approach in identifying TS-miRNAs epigenetically silenced in MM by treating MM cells with pharmacological compounds that reverse the silencing process. We show that hypomethylation treatment induced consistent upregulation of miRNAs in 4 HMCLs with distinct genetic background. These miRNAs include known TS-miRNAs silenced by hypermethylation, such as miR-192, miR-194 and miR-215 (listed in Supplementary Table S8). At the same time, many other miRNAs were unmasked, including miR-125a-3p, miR-155, miR-135a*, miR-198, miR-200c, miR-188-5p, miR-630, miR-663 and miR-483-5p. These miRNAs were either downregulated in MM patients, or had tumor suppressor functions in other cancers as reported previously. These were therefore selected for further analysis.

Human myeloma cell lines are mostly representative of advance disease and those from extramedullary sites. It is not clear whether these miRNAs were methylated in newly diagnosed myeloma. Therefore, we investigated the methylation status of the 9 candidate TS-miRNAs in 17 clinical myeloma samples consisting of 16 newly diagnosed and 1 relapsed case, comparing against 5 normal controls. Methylation array revealed DNA hypermethylation of these candidate TS-miRNAs in myeloma patients, suggesting that hypermethylation may contribute to their suppression in the clinical setting. Methylation of miR-155 is also identified in another recent study, and higher miR-155 level is associated with longer survival.[30]

MiR-155 is known to have oncogenic properties in B cell lymphomas [31] and other cancers.[32, 33] At the same time, it is also reported that miR-155 acted as tumor suppressor in cancers such as gastric cancer,[34] ovarian cancer [35] and melanoma,[36] suggesting that miR-155 is a multifunctional miRNA depending on cancer system. Consistent with its context-dependent function, In myeloma, ectopic expression of miR-155 reduced cell viability in MM1S and OPM2 cells but not H929 and 8226 cells. Moreover, we report tumor suppressor functions of miR-198, miR-135a*, miR-200c, miR-663 and miR-483-5p in myeloma, that they reduced cell viability, migration and colony formation. These miRNAs are predicted to target oncogenes such as CCND1, BCL9, HGF, and WHSC1. CCND1 is a positive cell cycle regulator that is frequently overexpressed in MM and other cancers;[37, 38] BCL9 confers enhanced proliferation and metastatic properties to cancer cells;[39] HGF enhances migration and survival of MM cells;[40] WHSC1 promotes cell cycle progression and adhesion of MM cells.[41, 42] Furthermore, we show that the predicted targets could potentially be of prognostic significance, as it contains signature that strongly associated with patient survival in several myeloma datasets.

Zhang et al have recently reported global miRNA suppression by methylation in myeloma, and specific miRNAs upregulated by another hypomethylating agent 5-aza-2′-deoxycytidine.[43] However, miRNAs discovered are different in the two studies. This apparent discrepancy may be explained by two factors. First, the platform used for miRNA expression profiling was different. We used oligonucleotide probe based Agilent array that covers all human miRNAs based on miRBase V16, while the other study used a different system and the miRBase coverage was not clear. Second, the other study used relapsed/refractory patient samples whereas our study used mostly newly diagnosed patient with higher number of samples. Investigation using standardized technology and larger patient cohorts may help addressing the discrepancy observed.

MiR-29b is another established tumor suppressor miRNA in myeloma.[44-47] The expression of miR-29b varies widely in MM, PCL and HMCL with reasons unclear.[48] We report that miR-29b can be consistently upregulated by 5′aza. (Supplementary Table S1) Moreover, increased methylation of mir-29b was observed in MM patients compared with normal plasma cells, (data not shown) suggesting that abnormal DNA methylation may be responsible for lower miR-29b level in subgroup of MM patients.

In summary, we show that pharmacological reversal of epigenetic silencing uncovered TS-miRNAs that are important for the survival and migration of malignant plasma cells, many for the first time. Furthermore, these epigenetically silenced TS-miRNA are clinically relevant as they are hypermethylated and suppressed in patient samples. Their predicted target genes are known to be relevant to the biology of myeloma and was found in our study to be important for prognosis. We acknowledge that false positives cannot be ruled out for miRNA targets predicted bioinformatically. Follow-up study on experimental validation and functional characterization of predicted miRNA targets is needed to elucidate the mechanism of action of TS-miRNAs.

Overall, our study provides further evidence that many of the deregulated miRNAs in myeloma are functionally relevant and epigenetic silencing is an important mechanism mediating their deregulation. This may have important therapeutic implications in two ways. One this provides a basis for the utility of epigenetic drugs in myeloma, both in derepressing epigenetically silenced tumor suppressor genes but perhaps more importantly because of the more extensive downstream effect, the depression of TS-miRNA. Second, with the advent of miRNA-based therapy, pharmacologic unmasking is an effective strategy in uncovering tumor suppressor miRNAs that may be novel therapeutic targets in myeloma.

## MATERIALS AND METHODS

### Cell culture, 5′aza treatment and miRNA overexpression

Human MM cell lines H929, MM1S, OPM2 and RPMI-8226 were maintained in RPMI 1640 (Life Technologies, Carlsbad, CA), supplemented with 10% fetal bovine serum (FBS), 1 mM L-glutamine, 100 units/ml penicillin and 100 mg/ml streptomycin. H929 was supplemented with 2-mercaptoethanol at a final concentration of 50μM. All cells were grown at 37°C in a humidified atmosphere with 5% CO_2_. All cells were gifts from Dr. Leif Bergsagel, Mayo Clinic, Scottsdale, Arizona, and authenticated before use. 5′azacytidine was purchased from Sigma-Aldrich (St. Louise, MO, USA) and was diluted just before use. For miRNA microarray, MM cells were treated with 5′aza at half maximal inhibitory concentration (IC_50_) which was determined with CalcuSyn software (Biosoft, Cambridge, United Kingdom) as previously described.[49] IC_50_ of 5′aza was 2μM for H929 and OPM2, and 1.5μM for MM1S and 8226 cells. Fresh medium containing 5′aza was added every 24 hours and cells were harvested at 96 hours after initial treatment. For miRNA functional study, miRNA mimics and negative control (Thermo Scientific, Waltham, Massachusetts, USA) were transfected into MM cells using DharmaFECT transfection reagent at a final concentration of 100nM. The efficiency of transfection was validated by TaqMan miRNA assay.

### RNA extraction and miRNA expression profiling

Total RNA, including small RNAs was extracted using MiRNeasy Kit (Qiagen, Valencia, CA). The purity of RNA was assessed by Nanodrop spectrophotometer (Thermo Scientific, Wilmington, DE) and the integrity was assessed by Agilent 2100 Bioanalyzer (Agilent Tech, Palo Alto, CA). All RNA samples showed high quality, without RNA degradation or DNA contamination. MiRNA expression profiling was using Agilent Human MiRNA Microarray (V16). Each array contained 60-mer probes representing 1205 human miRNAs from the miRBase (Version 16.0). Details of experimental procedure and data analysis were described in supplementary methods. Complete raw and normalized microarray data and their MIAME compliant metadata have been deposited at Gene Expression Omnibus (GEO) database under the accession number GSE53850.

### TaqMan miRNA assay

Real-Time RT-PCR quantification of mature miRNAs were carried out using TaqMan miRNA assay as previously described [50] using ABI 7300 Real-Time PCR system. (Applied Biosystems) U6 snRNA were used for normalization. All experiments were performed in triplicate. Relative expression was calculated using the comparative Ct method.

### Methylation profiling of patient samples

BM aspirates were obtained from 17 MM cases (16 newly diagnosed and 1 relapsed) with informed consent in accordance with the Declaration of Helsinki, and all studies were approved by National University of Singapore Institutional Review Board. CD138+ PCs were purified as described previously.[51] DNA extraction was done using QIAamp Mini kit (Qiagen). 5 normal control DNA from CD138+ PCs were provided by Dr. Rafael Fonseca and Dr. Gregory J Ahmann from Mayo Clinic, Arizona. DNA methylation data were generated using the Illumina Infinium Meth450K platform as per manufacturer's instructions. Illumina iDat files were imported in Partek Genomic Suite 6.0 for SWAN normalization [52] and subsequent analysis. Methylation data was presented as *β* values, with 0 indicating 0% DNA methylation and *β* values of 1 indicating 100% DNA methylation.

### miRNA target prediction

The target genes of the leading candidate TS-miRNAs were predicted using two online databases miRecords [53] and miRWalk.[54] These two databases integrated the prediction result of different miRNA target prediction algorithms including DIANA-microT, miRanda, PicTar, TargetScan, RNAhybrid, miRDB, RNA22, PITA, MicroInspector, NBmiRTar, miRWalk and miTarget. For each miRNA, targets commonly predicted by at least half of all available algorithms were selected for further analysis.

### Viability assay

The number of viable cells was determined using CellTiter-Glo Luminescent Cell Viability Assay (Promega, Madison MI, USA). Cells were seeded into 96-well plates at a density of 20,000 cells/well in 50-100ul culture medium. CellTiter-Glo reagent were added at 1:1 volume ratio. The plate was placed on a rocking platform for 2 minutes, and incubated at room temperature for 10 minutes before the luminescence signal was read in a plate reader (Tecan Infinite M200, Männedorf, Switzerland). Luminescence readings were background subtracted and normalized to control wells. Each experiment is performed with a minimum of three replicates.

### Migration assay

MM cells were diluted in RPMI 1640 without FBS in a ThinCert™ 8μM cell culture insert (Greiner bio-one, Frickenhausen, Germany) at a concentration of 10,000 cells per insert. The insert were placed into a well of a 24-well plate containing 750μl of full media containing FBS, and incubated in a 37°C in a humidified atmosphere with 5% CO_2_ for 18 hours. The inserts were removed and the cells that have migrated into the lower chamber were counted. Each experiment was performed in triplicate.

### Colony formation assay

Cells were diluted and mixed thoroughly in Methocult Methylcellulose media (Stemcell Technologies #4435, Vancouver, Canada) at a concentration of 5000 to 8000 cells per 2ml of Methocult media. It was then dispensed into the wells of 24-well plates at 0.5ml per well and incubated in a 37°C, humidified incubator with 5% CO_2_ for 1-2 weeks before colony counting. Each experiment was performed in triplicate.

### Statistical analysis

Two-tailed Mann Whitney U test was used to determine statistical significance between two groups of categorical variables. Cox regression was used to compare survival in groups with different risk score (RS) values. We also used Student's t-test, when appropriate. P-values less than 0.05 were considered to indicate statistically significant difference.

### Methylation-Specific PCR (MSP)

Detailed protocol is available in Supplementary Methods.

## SUPPLEMENTARY MATERIAL FIGURES AND TABLES


