# Supplementary material for: Genome-wide pharmacologic unmasking identifies tumor suppressive microRNAs in multiple myeloma
Source: Oncotarget. 2015 Jul 3;6(28):26508–18. doi: 10.18632/oncotarget.4769 (PMC4694918; doi:10.18632/oncotarget.4769)
Supplement: Supplementary file 1 [file oncotarget-06-26508-s001.pdf]

Genome-wide pharmacologic unmasking identifies tumor suppressive microRNAs in multiple myeloma

Supplementary Material

MiRNA Expression Profiling and Data Processing

MicroRNA expression was profiled using Agilent Human MiRNA Microarray (V16). Each array contained 60-mer probes representing 1205 human and 144 human viral miRNAs from the miRBase (Version 16.0). Array experiment was carried out using Agilent miRNA system protocol v2.4 as per manufacturer's instructions. Briefly, each RNA sample were labeled with Cyanine3-pCp and hybridized to the Agilent human miRNA microarray using the miRNA Complete Labeling and Hyb Kit (Agilent p/n 5190-0456). The slide was washed using Gene Expression Wash Buffer Kit (Agilent p/n 5188-5327), and then scanned using an Agilent DNA microarray scanner (G2565CA). Array QC was examined using two parameters, namely #FeatureNonUnif and %CV for replicated probes. Higher value of #FeatureUnonUnif indicates less uniformity of the distribution of signals, and higher value of %CV for replicated probes indicates lower reproducibility of signal across the array and lower signal-to-noise ratio. All samples passed the QC matrix and showed good data quality (#FeatureNonUnif <0.1% and %CV of replicated probes <15). The array QC reports, as well as miRNA expression data was extracted from the scanned image using Agilent Feature Extraction Software 10.7.3.1. Background subtracted, outlier rejected expression data was imported into GeneSpring GX12 and normalized using quantile method. Filtering on flag and expression was then carried out to exclude non detected miRNAs. Volcano plot was then used to calculate fold change. P-value was calcuated using the software default moderated T-Test with multiple testing correction (Benjamini-Hochberg method). Complete raw and normalized microarray data and their MIAME compliant metadata have been deposited at GEO (www.ncbi.nlm.nih.gov/geo) under the accession number GSE53850.

Methylation-Specifc PCR (MSP)

DNA extraction and bisulfite conversion were performed using Qiagen DNeasy Kit and EpiTect Bisulfite Kit respectively (Qiagen, Valencia, CA). Methylation-Specific PCR (MSP) primers were designed using Methyl Primer Express (Applied Biosystems, Foster City, CA) and the PCR reactions with final reaction volume of 25ul each were assembled with Invitrogen Platinum PCR Supermix (Thermo Fisher Scientific, Waltham, Massachusetts, USA), forward and reverse primers at a final concentration of 0.2uM each, and 50ng bisulfite-converted DNA. The PCR reactions were performed in a thermocycler under the following conditions: 94°C for 2 minutes; 36 cycles of: 94°C for 30 seconds, (primer annealing temperature) for 30 seconds, 72°C for

1 minute; and finally a final extension for 5 minutes at 72°C. Equal volume of PCR product for each sample was run on 2% w/v agarose gel and the image was obtained using biorad GelDoc XR+ imaging system. The intensity is quantified using ImageJ.[1] The primer sequences and annealing temperatures are listed in Supplementary Table 9.

MiRNA Localization and CpG Island Identification

The genomic localization of miRNAs were investigated using UCSC genome browser under Genome Reference Consortium Human Build 37 (GRCh37/hg19). CpG islands were predicted by the CpG island track in UCSC or by Methyl Primer Express software using DNA sequences 10 kilobase upstream and downstream of the pre-miRNA sequence, using the following three criteria: 1. GC content of 50% or higher; 2. Length greater than 200bp; 3. Ratio greater than 0.6 of observed number of CG dinucleotides to the expected number on the basis of the number of Gs and Cs in the segment.[2]

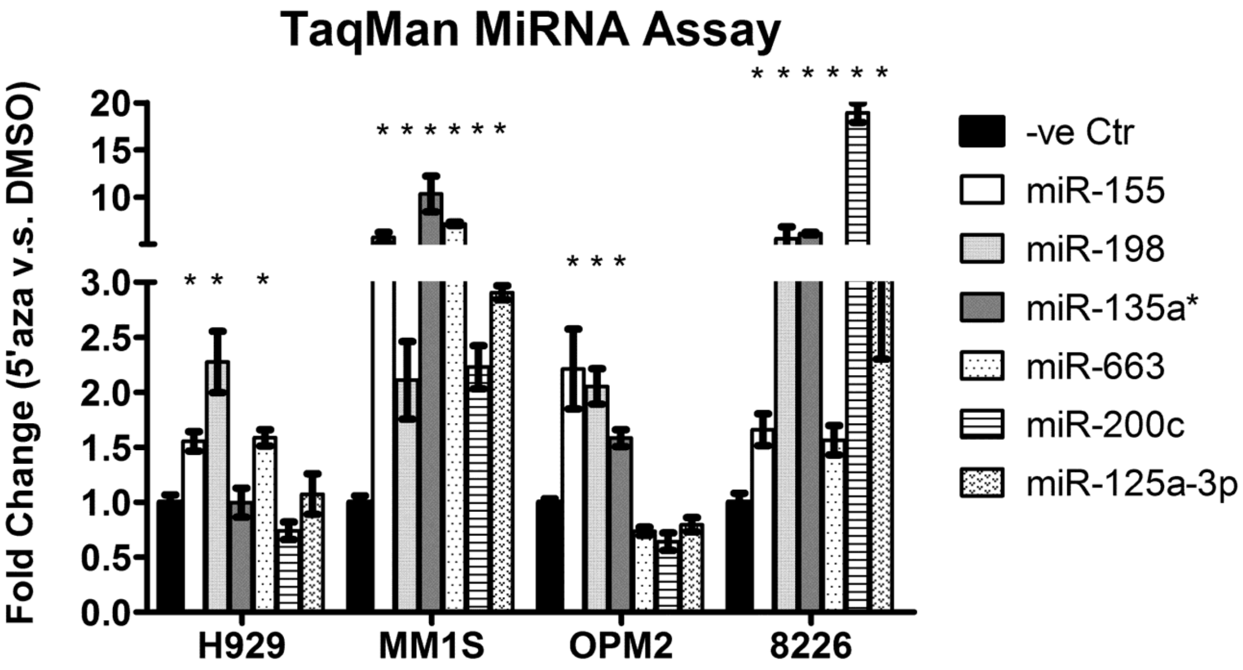

Supplementary Figure S1. Validation of upregulation after 5’azacytidine treatment for six miRNAs by Taqman miRNA assay

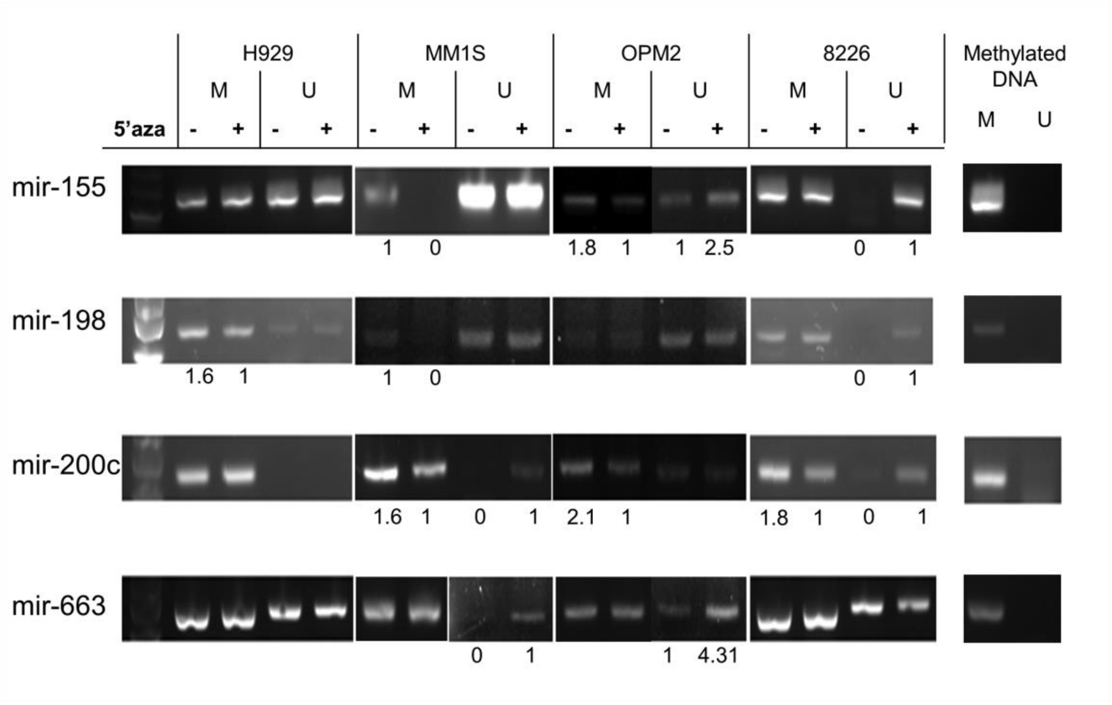

Supplementary Figure S2. Methylation-Specific PCR of representative leading candidate TS-miRNAs. The intensity of bands were quantified using ImageJ as per user manual of the software.

Supplementary Table S1. Result of miRNA microarray: 74 miRNAs commonly upregulated by 5’aza, sorted by chromosomal location

Supplementary Table S2. Methylation status of miRNA-associated CpG sites in MM patients and healthy controls. P-value was calculated using T-Test comparing beta value of patients and normal controls.

Supplementary Table S3. 215 Predicted miRNA targets that are also overexpressed in Myeloma patients compared with normal plasma cells (UAMS dataset GSE2658, cutoff q-value 0.005)

Supplementary Table S4. Upregulated genes in MM that are predicted to be the target of leading candidate TS-miRNAs, and their relevance in cancer in current literature

Supplementary Table S5. GSEA analysis, using the 215 predicted miRNA targets that are also upregulated in MM patients

Supplementary Table S6. KEGG Pathway Analysis of the predicted targets of leading candidate TS-miRNAs

Supplementary Table S7. 33 genes independently associated with survival in UAMS dataset are predicted to be targeted by leading candidate TS-miRNAs

Supplementary Table S8. Fold change after 5'aza treatment for known TS-miRNAs silenced by DNA methylation

Supplementary Table S9. Details of Methylation Specific PCR primers.

References

1. Schneider CA, Rasband WS and Eliceiri KW. NIH Image to ImageJ: 25 years of image analysis. Nat Methods. 2012; 9(7):671-675.

2. Gardiner-Garden M and Frommer M. CpG islands in vertebrate genomes. Journal of molecular biology. 1987; 196(2):261-282.

Supplementary Table S1. Result of miRNA microarray: 74 miRNAs commonly upregulated by 5'aza, sorted by chromosomal location. P-value was calculated by T-Test comparing the control group and 5'aza treated group in all four cell lines. Multiple Testing Correction was applied using Benjamini-Hochberg method. MiRNAs 29b,192,194 and 215 were previously reported tumor suppressors in MM (highlighted in hold and italic font); miRNAs within each orange box resided in the same miRNA cluster; miRNAs 135a*, 198, 200c, 125a-3p, 188-5p, and 155 were previously reported to be downregulated in myeloma patients whereas miRNAs mir-630, 663 and 483-5p were reported to have tumor suppressor properties in other cancers. (highlighted in green)
n.d.: not detected

	H929		MM1S		OPM2		8226							
systematic_name	fold change	regul ation	fold change	regul ation	fold change	regul ation	fold change	regul ation	p-value with FDR	Chro moso me	start	stop	mirbase accessi	str an d
hsa-miR-1290	1.64	up	141.58	up	2.41	up	2.07	up	0.235	chr1	19223635	19223619	MIMAT0005880	+
hsa-miR-30e*	7.28	up	58.11	down	1.64	up	1.57	down	0.777	chr1	40992676	40992693	MIMAT0000693	-
hsa-miR-4257	46.41	up	8.98	up	14.63	up	9.21	up	0.014	chr1	150524468	150524480	MIMAT0016878	-
hsa-miR-765	9.47	up	42.69	up	7.42	up	90.07	up	0.014	chr1	156906011	156905995	MIMAT0003945	+
hsa-miR-215	4.66	up	n.d.		1.75	up	n.d.		0.746	chr1	220291241	220291222	MIMAT0000272	+
hsa-miR-194	15.97	up	n.d.		2.50	up	1.81	up	0.569	chr1	220291534	220291519	MIMAT0000460	+
hsa-miR-1471	8.45	up	171.20	up	73.87	up	44.76	up	0.014	chr2	232756973	232756960	MIMAT0007349	+
hsa-miR-1246	3.27	up	15.83	up	6.25	up	15.86	up	0.138	chr2	177465736	177465722	MIMAT0005898	+
hsa-miR-3679-5p	1.70	up	7.00	up	1.66	up	5.55	up	0.016	chr2	134884711	134884723	MIMAT0018104	-
hsa-miR-135a*	55.11	up	5.36	up	10.97	up	37.37	up	0.022	chr3	52328311	52328300	MIMAT0004595	+
hsa-miR-4271	112.82	up	100.81	up	15.27	up	75.61	up	0.008	chr3	49311595	49311609	MIMAT0016901	-
hsa-miR-198	n.d.		13.22	up	n.d.		14.41	up	0.156	chr3	120114541	120114527	MIMAT0000228	+
hsa-miR-4270	146.39	up	344.51	up	2.09	up	5.51	up	0.042	chr3	15537775	15537764	MIMAT0016900	+
hsa-miR-1224-5p	n.d.		47.42	up	n.d.		8.21	up	0.182	chr3	183959202	183959211	MIMAT0005458	-
hsa-miR-1973	n.d.		3.85	up	n.d.		1.90	up	0.374	chr4	117220909	117220924	MIMAT0009448	-
hsa-miR-572	n.d.		6.28	up	n.d.		2.42	up	0.069	chr4	11370519	11370530	MIMAT0003237	-
hsa-miR-575	1.66	up	6.31	up	n.d.		3.52	up	0.071	chr4	83674568	83674552	MIMAT0003240	+
hsa-miR-1305	49.46	up	n.d.		55.62	up	50.77	up	0.040	chr4	183090500	183090517	MIMAT0005893	-
hsa-miR-4274	47.35	up	45.79	up	n.d.		n.d.		0.150	chr4	7461824	7461835	MIMAT0016906	-
hsa-miR-3945	n.d.		48.81	up	n.d.		8.15	up	0.183	chr4	185772211	185772193	MIMAT0018361	+
hsa-miR-3141	51.95	up	120.07	up	65.53	up	100.53	up	0.000	chr5	153975599	153975587	MIMAT0015010	+
hsa-miR-4281	2.65	up	9.16	up	1.87	up	4.13	up	0.014	chr5	176056491	176056481	MIMAT0016907	+
hsa-miR-874	1.97	up	1.51	up	n.d.		95.04	up	0.260	chr5	136983328	136983317	MIMAT0004911	+
hsa-miR-1275	1.55	up	4.16	up	1.71	up	3.69	up	0.014	chr6	33967782	33967770	MIMAT0005929	+
hsa-miR-219-5p	13.82	up	n.d.		8.85	up	n.d.		0.520	chr6	33175632	33175652	MIMAT0000276	-
hsa-miR-1202	n.d.		2.65	up	n.d.		1.75	up	0.213	chr6	156267952	156267963	MIMAT0005865	-
hsa-miR-877*	4.49	down	37.48	up	3.67	down	33.94	up	0.352	chr6	30552182	30552194	MIMAT0004950	-
hsa-miR-29b	1.90	up	1.68	up	1.66	up	n.d.		0.183	chr1	207975861	207975841	MIMAT0000100	+
hsa-miR-29a	2.20	up	2.63	up	1.61	up	n.d.		0.113	chr7	130561568	130561550	MIMAT0000086	+

hsa-miR-29a*	3.20	up	n.d.		2.22	up	n.d.		0.202	chr7	130561530	130561510	MIMAT0004503	+
hsa-miR-3610	n.d.		60.98	up	n.d.		7.70	up	0.190	chr8	117887027	117887017	MIMAT0017987	+
hsa-miR-1207-5p	2.05	up	8.69	up	2.38	up	4.41	up	0.014	chr8	129061414	129061425	MIMAT0005871	-
hsa-miR-151-5p	39.18	up	n.d.		14.84	up	n.d.		0.161	chr8	141742693	141742678	MIMAT0004697	+
hsa-miR-3911	n.d.		61.44	up	5.09	up	49.39	up	0.111	chr9	130452998	130452986	MIMAT0018185	+
hsa-miR-2861	n.d.		7.13	up	n.d.		3.20	up	0.058	chr9	130548259	130548268	MIMAT0013802	-
hsa-miR-3621	n.d.		7.90	up	4.90	up	n.d.		0.169	chr9	140063672	140063661	MIMAT0018002	+
hsa-miR-3663-3p	3.26	up	326.24	up	7.00	up	3.44	up	0.054	chr10	118927268	118927259	MIMAT0018085	+
hsa-miR-1915	2.19	up	5.27	up	1.56	up	2.36	up	0.018	chr10	21785556	21785547	MIMAT0007892	+
hsa-miR-4298	2.33	up	130.88	up	7.80	up	120.87	up	0.014	chr11	1880725	1880713	MIMAT0016852	+
hsa-miR-192	n.d.		3.16	up	1.55	up	n.d.		0.775	chr11	64658652	64658635	MIMAT0000222	+
hsa-miR-483-5p	2.60	up	297.01	up	131.91	up	343.55	up	0.014	chr11	2155392	2155379	MIMAT0004761	+
hsa-miR-3656	n.d.		1.89	up	n.d.		1.71	up	0.091	chr11	118889708	118889718	MIMAT0018076	-
hsa-miR-200c	n.d.		36.26	up	n.d.		55.71	up	0.151	chr12	7072914	7072927	MIMAT0000617	-
hsa-miR-141	n.d.		2.30	up	n.d.		200.34	up	0.483	chr12	7073324	7073339	MIMAT0000432	-
hsa-miR-3652	38.75	up	122.74	up	8.65	up	n.d.		0.045	chr12	104324206	104324220	MIMAT0018072	-
hsa-miR-3665	1.78	up	3.70	up	n.d.		1.61	up	0.039	chr13	78272188	78272180	MIMAT0018087	+
hsa-miR-134	119.61	up	205.21	up	108.34	up	148.14	up	0.000	chr14	101521041	101521052	MIMAT0000447	-
hsa-miR-345	n.d.		2.06	up	3.84	down	4.55	up	0.759	chr14	100774222	100774234	MIMAT0000772	-
hsa-miR-630	2.63	up	50.31	up	2.72	up	7.14	up	0.014	chr15	72879624	72879639	MIMAT0003299	-
hsa-miR-1268	1.55	up	3.95	up	1.73	up	1.83	up	0.022	chr15	22513250	22513239	MIMAT0005922	+
hsa-miR-762	2.75	up	7.40	up	1.58	up	3.43	up	0.020	chr16	30905283	30905293	MIMAT0010313	-
hsa-miR-1225-5p	1.66	up	3.86	up	n.d.		3.65	up	0.017	chr16	2140217	2140208	MIMAT0005572	+
hsa-miR-320c	n.d.		3.37	up	1.83	up	3.31	up	0.014	chr18	19263525	19263539	MIMAT0005793	-
hsa-miR-125a-3p	35.19	up	126.42	up	54.71	up	102.25	up	0.001	chr19	52196565	52196580	MIMAT0004602	-
hsa-miR-150*	110.54	up	65.50	up	n.d.		6.88	up	0.032	chr19	50004113	50004102	MIMAT0004610	+
hsa-miR-371-5p	153.60	up	132.56	up	51.41	up	47.70	up	0.001	chr19	54290942	54290953	MIMAT0004687	-
hsa-miR-642b	2.14	up	6.05	up	2.12	up	3.92	up	0.014	chr19	46178257	46178245	MIMAT0018444	+
hsa-miR-1181	175.07	up	135.55	up	n.d.		74.92	up	0.025	chr19	10514167	10514157	MIMAT0005826	+
hsa-miR-638	n.d.		8.01	up	n.d.		3.17	up	0.062	chr19	10829107	10829119	MIMAT0003308	-
hsa-miR-3195	n.d.		2.69	up	n.d.		2.44	up	0.029	chr20	60639874	60639884	MIMAT0015079	-
hsa-miR-663	107.25	up	445.96	up	2.01	up	2.90	up	0.036	chr20	26188857	26188846	MIMAT0003326	+
hsa-miR-3196	n.d.		4.17	up	1.61	up	1.75	up	0.034	chr20	61870146	61870157	MIMAT0015080	-
hsa-miR-1914*	n.d.		46.65	up	n.d.		7.87	up	0.183	chr20	62572888	62572877	MIMAT0007890	+
hsa-miR-3648	106.01	up	253.03	up	8.18	up	183.05	up	0.014	chr21	9825868	9825879	MIMAT0018068	-
hsa-miR-4327	63.05	up	68.73	up	n.d.		n.d.		0.149	chr21	31747640	31747628	MIMAT0016889	+
hsa-miR-155	8.52	up	2.60	up	n.d.		n.d.		0.673	chr21	26946302	26946317	MIMAT0000646	-
hsa-miR-3198	57.76	up	2.18	up	58.25	up	70.18	up	0.014	chr22	18247015	18247002	MIMAT0015083	+
hsa-miR-3667-5p	n.d.		150.66	up	3.39	up	162.98	up	0.078	chr22	49937070	49937055	MIMAT0018089	+
hsa-miR-718	52.64	up	148.56	up	81.48	up	42.57	up	0.001	chrX	153285432	153285423	MIMAT0012735	+
hsa-miR-424*	1.74	up	571.60	up	n.d.		109.89	up	0.056	chrX	133680711	133680699	MIMAT0004749	+
hsa-miR-513a-5p	1.67	up	1.83	up	n.d.		1.57	up	0.459	chrX	146295034	146295019	MIMAT0002877	+
hsa-miR-513c	3.21	up	2.94	up	n.d.		n.d.		0.180	chrX	146271256	146271239	MIMAT0005789	+

hsa-miR-513b	2.76	up	2.46	up	2.82	down	5.04	down	0.857	chrX	146280596	146280579	MIMAT0005788	+
hsa-miR-188-5p	2.67	up	618.70	up	2.84	up	6.17	up	0.067	chrX	49768132	49768143	MIMAT0000457	-

Supplementary Table S2. Methylation status of miRNA-associated CpG sites in MM patients and healthy controls. P-value was calculated using T-Test comparing beta value of patients and normal controls.

Pre-miRNA	CpG ID	Position	No.of MM patients with Δbeta more than 0.1 of average beta value of normal controls	Average beta value of patients	Average beta value of normal control	Δbeta value	p-value	Position_Comment
mir-125a	cg19818998	Downstream	6	0.358	0.046	0.312	0.004	S_Shore of CpG island
mir-155	cg00565412	Upstream of host gene (mir155hg)	4	0.780	0.496	0.284	0.055	N_Shore of CpG island; near TSS of host gene
mir-135a-1	cg22626659	Upstream	11	0.573	0.304	0.270	0.004	Enriched in H3K27Ac mark (marker for active transcription)
mir-135a-2	cg03782861	Upstream	5	0.599	0.256	0.343	0.038	near TSS of host gene
mir-188	cg03662899	Upstream	6	0.853	0.621	0.233	0.004	near TSS of mir-188
mir-198	cg04865442	Upstream	7	0.854	0.535	0.319	0.000	N_Shore of CpG island; near TSS of host gene
mir-200c	cg00366413	Upstream	10	0.752	0.514	0.238	0.000	CpG rich sequence near TSS of mir-200c
mir-483	cg23905216	Upstream	8	0.617	0.355	0.262	0.010	CpG island
mir-630	cg15188939	Upstream	10	0.691	0.388	0.303	0.014	Intergenic of host gene
mir-663	cg01521987	Upstream	13	0.729	0.227	0.502	0.000	CpG island

Supplementary Table S3. Predicted miRNA targets that are also overexpressed in Myeloma patients compared with normal plasma cells (UAMS dataset GSE2658, cutoff q-value 0.005)

miRNA ID	Predicted Target	Fold increase in MM	Adjusted P-value
hsa-miR-200c	ATXN1	1.554	1.934E-05
hsa-miR-188-5p	ATXN1	1.554	1.934E-05
hsa-miR-630	ESRRG	2.689	0.000E+00
hsa-miR-200c	ESRRG	2.689	0.000E+00
hsa-miR-200c	KLF13	1.784	0.000E+00
hsa-miR-188-5p	KLF13	1.784	0.000E+00
hsa-miR-198	PTEN	1.832	0.000E+00
hsa-miR-188-5p	PTEN	1.832	0.000E+00
hsa-miR-135a*	PTEN	1.832	0.000E+00
hsa-miR-155	AKAP10	1.602	0.000E+00
hsa-miR-630	AKAP10	1.602	0.000E+00
hsa-miR-135a*	BACE1	1.840	0.000E+00
hsa-miR-125a-3p	CYCS	1.920	0.000E+00
hsa-miR-200c	DCUN1D1	1.644	0.000E+00
hsa-miR-135a*	DCUN1D1	1.644	0.000E+00
hsa-miR-630	DOCK8	1.690	1.470E-05
hsa-miR-188-5p	FAM126B	1.578	6.360E-06
hsa-miR-135a*	FAM126B	1.578	6.360E-06
hsa-miR-198	FAM55C	1.614	2.594E-05
hsa-miR-200c	FBXO33	1.865	0.000E+00
hsa-miR-155	FBXO33	1.865	0.000E+00
hsa-miR-200c	GLCCI1	2.879	6.568E-06
hsa-miR-155	GLCCI1	2.879	6.568E-06
hsa-miR-135a*	KIAA0323	1.635	1.969E-05
hsa-miR-155	KIAA1267	1.867	1.470E-05
hsa-miR-135a*	KIAA1267	1.867	1.470E-05
hsa-miR-125a-3p	LARP4	1.519	0.000E+00
hsa-miR-200c	MAP2	2.262	0.000E+00
hsa-miR-135a*	MAP2	2.262	0.000E+00
hsa-miR-200c	MATR3	1.838	0.000E+00
hsa-miR-155	MATR3	1.838	0.000E+00
hsa-miR-200c	MBNL1	1.666	0.000E+00
hsa-miR-188-5p	MBNL1	1.666	0.000E+00
hsa-miR-125a-3p	MRPS25	1.606	4.483E-06
hsa-miR-200c	MXD4	1.575	9.459E-06
hsa-miR-200c	NR3C1	1.978	0.000E+00
hsa-miR-200c	NUFIP2	1.524	0.000E+00
hsa-miR-135a*	NUFIP2	1.524	0.000E+00
hsa-miR-155	PCDH9	2.184	0.000E+00
hsa-miR-188-5p	PCDH9	2.184	0.000E+00
hsa-miR-125a-3p	PGPEP1	1.980	0.000E+00

hsa-miR-135a*	PKD2	1.596	0.000E+00
hsa-miR-188-5p	PML	1.518	0.000E+00
hsa-miR-188-5p	RICTOR	1.581	4.087E-06
hsa-miR-155	RICTOR	1.581	4.087E-06
hsa-miR-200c	SCAMP1	2.031	0.000E+00
hsa-miR-135a*	SCAMP1	2.031	0.000E+00
hsa-miR-188-5p	SERBP1	1.585	0.000E+00
hsa-miR-198	SNX1	2.296	0.000E+00
hsa-miR-200c	TSGA14	1.672	0.000E+00
hsa-miR-155	TSGA14	1.672	0.000E+00
hsa-miR-630	UPF1	2.082	0.000E+00
hsa-miR-135a*	VGLL4	1.554	0.000E+00
hsa-miR-125a-3p	ZFP3	1.737	3.227E-05
hsa-miR-188-5p	ZNF197	1.611	6.568E-06
hsa-miR-483-5p	ABCF2	2.618	0.000E+00
hsa-miR-135a*	ABI3BP	2.134	0.000E+00
hsa-miR-135a*	ACVR1C	1.635	0.000E+00
hsa-miR-188-5p	ACVR2A	1.547	0.000E+00
hsa-miR-125a-3p	AGPAT3	1.808	0.000E+00
hsa-miR-135a*	AGTPBP1	1.603	5.453E-06
hsa-miR-630	AIG1	1.908	0.000E+00
hsa-miR-135a*	ALS2	1.709	0.000E+00
hsa-miR-125a-3p	AMACR	1.534	0.000E+00
hsa-miR-135a*	ANKIB1	1.972	1.635E-05
hsa-miR-135a*	ANKRD10	1.515	4.760E-04
hsa-miR-200c	ANKRD28	2.470	0.000E+00
hsa-miR-135a*	ANKRD50	1.545	2.003E-05
hsa-miR-198	ARHGAP19	1.805	0.000E+00
hsa-miR-200c	ARHGDIA	1.883	1.969E-05
hsa-miR-155	ARID2	1.708	6.568E-06
hsa-miR-200c	ARID4B	1.521	6.360E-06
hsa-miR-200c	ARIH2	1.555	0.000E+00
hsa-miR-200c	ASXL1	2.245	0.000E+00
hsa-miR-630	ATG12	1.573	1.292E-06
hsa-miR-200c	BAP1	1.666	3.032E-05
hsa-miR-200c	BAZ2B	1.774	0.000E+00
hsa-miR-188-5p	BCL9	2.321	0.000E+00
hsa-miR-200c	BICD2	1.731	6.688E-07
hsa-miR-135a*	BMPR2	1.697	0.000E+00
hsa-miR-125a-3p	BRCA1	1.908	0.000E+00
hsa-miR-188-5p	BRCC3	1.730	0.000E+00
hsa-miR-200c	C10orf6	1.743	1.547E-05
hsa-miR-200c	C16orf72	1.552	1.129E-05
hsa-miR-188-5p	C1orf25	1.847	0.000E+00
hsa-miR-630	C1orf96	1.512	5.137E-05
hsa-miR-630	C2orf37	1.523	0.000E+00
hsa-miR-663	C7orf26	2.565	0.000E+00

hsa-miR-188-5p	C7orf38	1.614	5.453E-06
hsa-miR-200c	CAMSAP1L1	2.285	0.000E+00
hsa-miR-135a*	CASC3	1.557	1.635E-05
hsa-miR-125a-3p	CASD1	1.550	2.594E-05
hsa-miR-198	CBS	1.720	4.087E-06
hsa-miR-155	CCND1	2.803	0.000E+00
hsa-miR-188-5p	CCNT2	1.598	0.000E+00
hsa-miR-630	CD200	1.891	0.000E+00
hsa-miR-155	CD47	1.520	0.000E+00
hsa-miR-135a*	CDC42SE1	2.021	0.000E+00
hsa-miR-155	CHD7	2.593	0.000E+00
hsa-miR-630	CLIC2	1.786	9.209E-05
hsa-miR-663	CNN2	1.568	1.272E-05
hsa-miR-135a*	CPEB2	1.718	0.000E+00
hsa-miR-630	CPEB4	2.048	0.000E+00
hsa-miR-188-5p	CREB3L2	1.710	2.604E-04
hsa-miR-155	CSNK1A1	1.537	2.523E-06
hsa-miR-155	CSNK1G2	1.658	0.000E+00
hsa-miR-188-5p	CUL4B	1.666	6.688E-07
hsa-miR-630	CYP51A1	1.554	4.087E-06
hsa-miR-135a*	DDX11	1.803	0.000E+00
hsa-miR-200c	E2F3	1.722	2.991E-04
hsa-miR-125a-3p	EIF5	1.590	0.000E+00
hsa-miR-200c	EIF5B	1.591	4.123E-05
hsa-miR-188-5p	ELOVL4	2.199	0.000E+00
hsa-miR-135a*	ENAH	1.741	0.000E+00
hsa-miR-125a-3p	FAHD1	1.553	3.073E-06
hsa-miR-135a*	FANCF	1.718	2.003E-05
hsa-miR-198	FANCL	1.529	4.087E-06
hsa-miR-198	FBXO22	1.722	0.000E+00
hsa-miR-200c	FHL1	1.616	4.123E-05
hsa-miR-663	FKBP8	2.015	7.839E-06
hsa-miR-200c	FLI1	1.916	4.483E-06
hsa-miR-200c	FNDC3B	1.844	3.032E-05
hsa-miR-125a-3p	FUT6	1.671	0.000E+00
hsa-miR-125a-3p	FXR1	1.659	6.568E-06
hsa-miR-200c	GABPA	1.872	0.000E+00
hsa-miR-188-5p	GLIS3	1.546	6.568E-06
hsa-miR-125a-3p	GMCL1	1.507	6.568E-06
hsa-miR-155	GNAS	1.589	3.351E-05
hsa-miR-630	GNB1	1.730	0.000E+00
hsa-miR-188-5p	H3F3B	2.027	1.995E-04
hsa-miR-188-5p	HDAC9	1.524	1.295E-04
hsa-miR-135a*	HGF	3.129	0.000E+00
hsa-miR-200c	HIC2	1.599	1.153E-04
hsa-miR-135a*	HLA-A	1.825	0.000E+00
hsa-miR-200c	HRB	1.577	4.087E-06

hsa-miR-198	IDS	2.041	0.000E+00
hsa-miR-188-5p	IL13RA1	1.656	6.568E-06
hsa-miR-188-5p	ILF3	1.718	0.000E+00
hsa-miR-135a*	IQGAP1	1.539	9.241E-05
hsa-miR-188-5p	ISG20L1	2.287	0.000E+00
hsa-miR-155	KBTBD2	1.521	0.000E+00
hsa-miR-155	KIAA1274	1.616	0.000E+00
hsa-miR-155	KPNA1	1.502	2.913E-04
hsa-miR-200c	LAMC1	2.146	0.000E+00
hsa-miR-188-5p	LAPTM4B	1.553	2.792E-04
hsa-miR-200c	LEPR	1.583	0.000E+00
hsa-miR-188-5p	LSM12	1.719	0.000E+00
hsa-miR-135a*	LYPLAL1	1.595	5.941E-06
hsa-miR-188-5p	MAP3K3	1.822	0.000E+00
hsa-miR-200c	MAP4K3	1.622	1.111E-05
hsa-miR-135a*	MAT2A	1.589	8.899E-05
hsa-miR-155	MECP2	1.831	0.000E+00
hsa-miR-188-5p	MEF2C	1.646	0.000E+00
hsa-miR-630	MEF2D	1.752	3.170E-05
hsa-miR-198	METTL9	1.559	0.000E+00
hsa-miR-125a-3p	MTAP	1.551	0.000E+00
hsa-miR-125a-3p	MTERFD2	1.713	4.087E-06
hsa-miR-200c	MTUS1	2.008	0.000E+00
hsa-miR-155	MYBL1	1.828	6.688E-07
hsa-miR-200c	NAP1L5	1.915	0.000E+00
hsa-miR-188-5p	NBEA	2.766	0.000E+00
hsa-miR-198	NDRG3	1.673	6.030E-04
hsa-miR-200c	NIPBL	2.126	0.000E+00
hsa-miR-135a*	NLK	1.691	1.292E-06
hsa-miR-125a-3p	NPTN	1.638	0.000E+00
hsa-miR-200c	NR2C2	1.532	3.227E-05
hsa-miR-200c	NUDT4	1.744	1.726E-04
hsa-miR-135a*	NUP160	1.758	0.000E+00
hsa-miR-188-5p	OAS3	1.983	4.087E-06
hsa-miR-200c	ORMDL3	1.883	2.054E-05
hsa-miR-200c	OXR1	1.671	2.162E-05
hsa-miR-188-5p	PARP16	2.380	0.000E+00
hsa-miR-200c	PCMTD1	1.756	0.000E+00
hsa-miR-200c	PDE5A	1.586	4.483E-06
hsa-miR-630	PELI1	3.173	0.000E+00
hsa-miR-155	PHF17	1.530	1.166E-04
hsa-miR-630	PIGK	1.563	1.090E-04
hsa-miR-200c	PIN1	2.207	0.000E+00
hsa-miR-198	POM121	1.656	0.000E+00
hsa-miR-200c	PPP1R10	1.759	0.000E+00
hsa-miR-200c	PPP2CA	1.511	6.427E-05
hsa-miR-200c	PRDM1	2.158	8.634E-05
hsa-miR-125a-3p	PRDM5	2.691	0.000E+00
hsa-miR-630	PRKCI	1.562	3.327E-05
hsa-miR-200c	PTPRZ1	1.536	4.016E-04
hsa-miR-135a*	PURA	1.858	0.000E+00
hsa-miR-198	RAPGEF4	1.593	4.087E-06
hsa-miR-188-5p	RBM8A	1.973	0.000E+00
hsa-miR-155	RCN2	1.694	0.000E+00

hsa-miR-200c	RFXDC2	1.602	6.568E-06
hsa-miR-135a*	RHOBTB3	1.722	0.000E+00
hsa-miR-155	RNF123	1.633	0.000E+00
hsa-miR-200c	SAPS3	2.056	0.000E+00
hsa-miR-155	SATB1	1.569	1.470E-05
hsa-miR-200c	SBF1	2.266	0.000E+00
hsa-miR-135a*	SENP2	1.964	5.453E-06
hsa-miR-135a*	SF3A3	1.507	0.000E+00
hsa-miR-188-5p	SLC12A2	1.571	0.000E+00
hsa-miR-630	SLC30A5	1.615	0.000E+00
hsa-miR-188-5p	SLC35A5	1.501	6.568E-06
hsa-miR-125a-3p	SMARCA2	2.009	0.000E+00
hsa-miR-155	SMARCA4	2.916	0.000E+00
hsa-miR-200c	SMARCAD1	1.556	4.854E-04
hsa-miR-630	SOCS2	1.615	6.688E-07
hsa-miR-630	ST3GAL6	3.289	0.000E+00
hsa-miR-135a*	SUZ12	1.686	3.073E-06
hsa-miR-200c	TBC1D22B	1.627	2.693E-05
hsa-miR-630	TDO2	1.577	0.000E+00
hsa-miR-155	TERF1	1.701	0.000E+00
hsa-miR-125a-3p	TEX261	1.827	0.000E+00
hsa-miR-135a*	TIMM22	2.238	0.000E+00
hsa-miR-200c	TMCC1	1.867	2.945E-05
hsa-miR-200c	TMEFF2	1.643	3.630E-06
hsa-miR-188-5p	TMEM128	1.546	1.470E-05
hsa-miR-200c	TOB1	1.664	0.000E+00
hsa-miR-630	TP53RK	1.599	1.015E-05
hsa-miR-200c	TRIM33	1.678	3.073E-06
hsa-miR-200c	TSC22D2	1.535	0.000E+00
hsa-miR-125a-3p	TTC14	2.327	4.087E-06
hsa-miR-200c	TUBB3	1.569	7.221E-05
hsa-miR-188-5p	USP47	1.751	0.000E+00
hsa-miR-198	VBP1	1.549	6.688E-07
hsa-miR-125a-3p	VISA	1.549	5.429E-04
hsa-miR-135a*	WDR42A	1.594	1.015E-05
hsa-miR-155	WEE1	1.504	0.000E+00
hsa-miR-188-5p	WHSC1	2.320	0.000E+00
hsa-miR-135a*	ZBTB38	2.230	0.000E+00
hsa-miR-188-5p	ZC3HAV1	1.642	3.032E-05
hsa-miR-630	ZDHHC21	1.560	0.000E+00
hsa-miR-200c	ZFAND6	1.915	0.000E+00
hsa-miR-155	ZMYM2	1.545	3.733E-04
hsa-miR-200c	ZNF532	1.980	0.000E+00
hsa-miR-630	ZNF605	1.634	0.000E+00
hsa-miR-630	ZNF770	1.654	1.111E-05

Supplementary Table S4. Upregulated genes in MM that are predicted to be the target of leading candidate TS-miRNAs, and their relevance in cancer in current literature

miRNA ID	Predicted Target	Fold increase in MM	Adjusted P-value	Relevance in cancer
hsa-miR-125a-3p	AGPAT3	1.81	0.000E+00	
hsa-miR-125a-3p	AMACR	1.53	0.000E+00	overexpressed in prostate cancer[1]
hsa-miR-125a-3p	BRCA1	1.91	0.000E+00	
hsa-miR-125a-3p	CASD1	1.55	2.594E-05	
hsa-miR-125a-3p	CYCS	1.92	0.000E+00	
hsa-miR-125a-3p	EIF5	1.59	0.000E+00	
hsa-miR-125a-3p	FAHD1	1.55	3.073E-06	
hsa-miR-125a-3p	FUT6	1.67	0.000E+00	highly expressed in metastatic colorectal cancer; involved in cell adhesion and migration[2]
hsa-miR-125a-3p	FXR1	1.66	6.568E-06	
hsa-miR-125a-3p	GMCL1	1.51	6.568E-06	
hsa-miR-125a-3p	LARP4	1.52	0.000E+00	
hsa-miR-125a-3p	MRPS25	1.61	4.483E-06	
hsa-miR-125a-3p	MTAP	1.55	0.000E+00	
hsa-miR-125a-3p	MTERFD2	1.71	4.087E-06	
hsa-miR-125a-3p	NPTN	1.64	0.000E+00	elevated expression correlates with metastasis of liver cancer[3]; overexpressed in breast cancer and promote growth and metastasis of cancer cells[4]
hsa-miR-125a-3p	PGPEP1	1.98	0.000E+00	
hsa-miR-125a-3p	PRDM5	2.69	0.000E+00	
hsa-miR-125a-3p	SMARCA2	2.01	0.000E+00	high expression is associated with lung cancer[5]
hsa-miR-125a-3p	TEX261	1.83	0.000E+00	
hsa-miR-125a-3p	TTC14	2.33	4.087E-06	
hsa-miR-125a-3p	VISA	1.55	5.429E-04	
hsa-miR-125a-3p	ZFP3	1.74	3.227E-05	
hsa-miR-135a*	ABI3BP	2.13	0.000E+00	
hsa-miR-135a*	ACVR1C	1.64	0.000E+00	
hsa-miR-135a*	AGTPBP1	1.60	5.453E-06	
hsa-miR-135a*	ALS2	1.71	0.000E+00	
hsa-miR-135a*	ANKIB1	1.97	1.635E-05	
hsa-miR-135a*	ANKRD10	1.51	4.760E-04	
hsa-miR-135a*	ANKRD50	1.54	2.003E-05	
hsa-miR-135a*	BACE1	1.84	0.000E+00	
hsa-miR-135a*	BMPR2	1.70	0.000E+00	
hsa-miR-135a*	CASC3	1.56	1.635E-05	
hsa-miR-135a*	CDC42SE1	2.02	0.000E+00	
hsa-miR-135a*	CPEB2	1.72	0.000E+00	
hsa-miR-135a*	DDX11	1.80	0.000E+00	overexpressed in primary and metastatic melanoma[6]
hsa-miR-135a*	ENAH	1.74	0.000E+00	associated with progression and metastasis in breast cancer[7, 8]
hsa-miR-135a*	FANCF	1.72	2.003E-05	silencing of FANCF potentiates

				mitroxantrone-mediated apoptosis in breast cancer cells[9]
hsa-miR-135a*	HGF	3.13	0.000E+00	Promotes viability and migration of myeloma cells[10, 11]
hsa-miR-135a*	HLA-A	1.83	0.000E+00	
hsa-miR-135a*	IQGAP1	1.54	9.241E-05	regulates cell migration[12, 13]; overexpressed in pancreatic cancer[14]; required for RAS-driven tumorigenesis[15]
hsa-miR-135a*	KIAA0323	1.64	1.969E-05	
hsa-miR-135a*	LYPLAL1	1.60	5.941E-06	
hsa-miR-135a*	MAT2A	1.59	8.899E-05	upregulated in HCC[16]
hsa-miR-135a*	NLK	1.69	1.292E-06	key regulator of proliferation and migration in gallbladder carcinoma cells[17];<br>upregulated in HCC and Inhibition of NLK reduced HCC tumor cell growth[18]
hsa-miR-135a*	NUP160	1.76	0.000E+00	
hsa-miR-135a*	PKD2	1.60	0.000E+00	
hsa-miR-135a*	PURA	1.86	0.000E+00	
hsa-miR-135a*	RHOBTB3	1.72	0.000E+00	
hsa-miR-135a*	SENP2	1.96	5.453E-06	
hsa-miR-135a*	SF3A3	1.51	0.000E+00	
hsa-miR-135a*	SUZ12	1.69	3.073E-06	promotes proliferation of epithelial ovarian cancer cells[19]
hsa-miR-135a*	TIMM22	2.24	0.000E+00	
hsa-miR-135a*	VGLL4	1.55	0.000E+00	associated with poor patient survival in pancreatic cancer[20]
hsa-miR-135a*	WDR42A	1.59	1.015E-05	
hsa-miR-135a*	ZBTB38	2.23	0.000E+00	
hsa-miR-155	ARID2	1.71	6.568E-06	
hsa-miR-155	CCND1	2.80	0.000E+00	overexpressed in MM and other cancers[21, 22]
hsa-miR-155	CD47	1.52	0.000E+00	activates migration in B-cells[23]; regulates tumor metastasis in MM to bone[24]; anti-CD47 antibody treatment inhibits MM cells growth[25]
hsa-miR-155	CHD7	2.59	0.000E+00	
hsa-miR-155	CSNK1A1	1.54	2.523E-06	high expression associated with poor prognosis in NSCLC[26]
hsa-miR-155	CSNK1G2	1.66	0.000E+00	
hsa-miR-155	GNAS	1.59	3.351E-05	amplification confers unfavorable PFS in epithelial ovarian cancer[27]
hsa-miR-155	KBTBD2	1.52	0.000E+00	
hsa-miR-155	KIAA1274	1.62	0.000E+00	
hsa-miR-155	KPNA1	1.50	2.913E-04	
hsa-miR-155	MECP2	1.83	0.000E+00	promotes cell proliferation in HCC[28]
hsa-miR-155	MYBL1	1.83	6.688E-07	
hsa-miR-155	PHF17	1.53	1.166E-04	
hsa-miR-155	RCN2	1.69	0.000E+00	
hsa-miR-155	RNF123	1.63	0.000E+00	
hsa-miR-155	SATB1	1.57	1.470E-05	associated with NPC

				progression[29]; Prostate cancer progression[30]
hsa-miR-155	SMARCA4	2.92	0.000E+00	prognostic marker and potential therapeutic target in breast cancer[31]
hsa-miR-155	TERF1	1.70	0.000E+00	
hsa-miR-155	WEE1	1.50	0.000E+00	high expression is associated with poor disease-free survival in malignant melanoma[32]
hsa-miR-155	ZMYM2	1.55	3.733E-04	fusion partner of FGFR1, fusion gene is oncogenic[33]
hsa-miR-155, has-miR-630	AKAP10	1.60	0.000E+00	higher expression in colorectal cancer and correlated with aggressiveness[34]
hsa-miR-155, hsa-miR-135a*	KIAA1267	1.87	1.470E-05	
hsa-miR-155, hsa-miR-188-5p	PCDH9	2.18	0.000E+00	
hsa-miR-188-5p	ACVR2A	1.55	0.000E+00	
hsa-miR-188-5p	BCL9	2.32	0.000E+00	promotes tumor progression by conferring enhanced proliferative, metastatic, and angiogenic properties to cancer cells[35, 36]
hsa-miR-188-5p	BRCC3	1.73	0.000E+00	
hsa-miR-188-5p	C1orf25	1.85	0.000E+00	
hsa-miR-188-5p	C7orf38	1.61	5.453E-06	
hsa-miR-188-5p	CCNT2	1.60	0.000E+00	
hsa-miR-188-5p	CREB3L2	1.71	2.604E-04	
hsa-miR-188-5p	CUL4B	1.67	6.688E-07	high expression associated with poor prognosis in colon cancer[37]; promotes cell proliferation, invasion, and tumorigenesis in vitro and in vivo; expression is markedly upregulated in various human cancers[38]
hsa-miR-188-5p	ELOVL4	2.20	0.000E+00	
hsa-miR-188-5p	GLIS3	1.55	6.568E-06	increased in glioblastoma[39]; overexpressed in ependymomas and associated with a poor outcome[40]
hsa-miR-188-5p	H3F3B	2.03	1.995E-04	
hsa-miR-188-5p	HDAC9	1.52	1.295E-04	higher expression associates with a lower 5-yr survival in paediatric ALL[41]
hsa-miR-188-5p	IL13RA1	1.66	6.568E-06	
hsa-miR-188-5p	ILF3	1.72	0.000E+00	promotes breast tumor progression by regulating sustained urokinase-type plasminogen activator expression[42]
hsa-miR-188-5p	ISG20L1	2.29	0.000E+00	
hsa-miR-188-5p	LAPTM4B	1.55	2.792E-04	upregulated in various human tumors and is oncogene[43] that predicts clinical outcome[44]; amplification contributes to chemo-resistance and recurrence of breast cancer[43]
hsa-miR-188-5p	LSM12	1.72	0.000E+00	
hsa-miR-188-5p	MAP3K3	1.82	0.000E+00	amplified in breast cancer,

				promotes formation and survival of breast cancer cells[45]
hsa-miR-188-5p	MEF2C	1.65	0.000E+00	upregulated in CML[46]; potential oncogene in T-ALL[47]; upregulated in AML with MLL gene disruptions and associates with homing and invasiveness of MLL/ENL leukemic cells[48]
hsa-miR-188-5p	NBEA	2.77	0.000E+00	fusion partner of PVT1 genes that highly expressed in subgroup of MM[49]
hsa-miR-188-5p	OAS3	1.98	4.087E-06	
hsa-miR-188-5p	PARP16	2.38	0.000E+00	
hsa-miR-188-5p	PML	1.52	0.000E+00	
hsa-miR-188-5p	RBM8A	1.97	0.000E+00	upregulated in lymph metastasis of cervical cancer[50]
hsa-miR-188-5p	SERBP1	1.59	0.000E+00	overexpressed in epithelial ovarian cancer cells and correlates with tumor stage[51]
hsa-miR-188-5p	SLC12A2	1.57	0.000E+00	regulates glioma cell migration[52]; meningionma invasion[53]
hsa-miR-188-5p	SLC35A5	1.50	6.568E-06	increased paclitaxel susceptibility with knockdown of SLC35A5[54]
hsa-miR-188-5p	TMEM128	1.55	1.470E-05	
hsa-miR-188-5p	USP47	1.75	0.000E+00	regulates cell growth and survival, potential oncogene[55]
hsa-miR-188-5p	WHSC1	2.32	0.000E+00	promotes cell cycle progression and adhesion of MM cells[56, 57]
hsa-miR-188-5p	ZC3HAV1	1.64	3.032E-05	
hsa-miR-188-5p	ZNF197	1.61	6.568E-06	
hsa-miR-188-5p, hsa-miR-135a*	FAM126B	1.58	6.360E-06	
hsa-miR-188-5p, hsa-miR-155	RICTOR	1.58	4.087E-06	promotes cell migration[58]; contribute to cisplatin resistance in ovarian cancer[59]
hsa-miR-198	ARHGAP1 9	1.80	0.000E+00	contribute to EMT and aggressive behavior in cancer cells[60]
hsa-miR-198	CBS	1.72	4.087E-06	
hsa-miR-198	FAM55C	1.61	2.594E-05	
hsa-miR-198	FANCL	1.53	4.087E-06	
hsa-miR-198	FBXO22	1.72	0.000E+00	
hsa-miR-198	IDS	2.04	0.000E+00	
hsa-miR-198	METTL9	1.56	0.000E+00	
hsa-miR-198	NDRG3	1.67	6.030E-04	upregulated in prostate cancer[61]; promotes prostate cancer cell growth[62]
hsa-miR-198	POM121	1.66	0.000E+00	
hsa-miR-198	RAPGEF4	1.59	4.087E-06	plays an important role in pancreatic cancer cell migration and invasion[63]; potential target for the suppression of melanoma cell migration[64]
hsa-miR-198	SNX1	2.30	0.000E+00	
hsa-miR-198	VBP1	1.55	6.688E-07	
hsa-miR-198, hsa-miR-188-5p, hsa-miR-135a*	PTEN	1.83	0.000E+00	

hsa-miR-200c	ANKRD28	2.47	0.000E+00	promotes cell migration by regulating focal adhesion formation[65]
hsa-miR-200c	ARHGDIA	1.88	1.969E-05	associated with metastasis in colon and prostate cancer[66]; downregulation of RhoGDIα was associated with significantly increased apoptosis and repressed cell viability in lung cancer cells[67]
hsa-miR-200c	ARID4B	1.52	6.360E-06	
hsa-miR-200c	ARIH2	1.55	0.000E+00	
hsa-miR-200c	ASXL1	2.25	0.000E+00	
hsa-miR-200c	BAP1	1.67	3.032E-05	
hsa-miR-200c	BAZ2B	1.77	0.000E+00	
hsa-miR-200c	BICD2	1.73	6.688E-07	
hsa-miR-200c	C10orf6	1.74	1.547E-05	
hsa-miR-200c	C16orf72	1.55	1.129E-05	
hsa-miR-200c	CAMSAP1 L1	2.29	0.000E+00	
hsa-miR-200c	E2F3	1.72	2.991E-04	oncogenic properties in breast and lung cancer[68, 69]
hsa-miR-200c	EIF5B	1.59	4.123E-05	
hsa-miR-200c	FHL1	1.62	4.123E-05	
hsa-miR-200c	FLI1	1.92	4.483E-06	contribute to pathogenesis of DLBCL[70]
hsa-miR-200c	FNDC3B	1.84	3.032E-05	oncogenic in liver cancer; induces EMT[71]
hsa-miR-200c	GABPA	1.87	0.000E+00	required for development of CML[72], maintenance and differentiation of HSC/progenitor cells[73]
hsa-miR-200c	HIC2	1.60	1.153E-04	
hsa-miR-200c	HRB	1.58	4.087E-06	
hsa-miR-200c	LAMC1	2.15	0.000E+00	associated with meningioma grades and could play a role in enhancing tumor invasion[74]
hsa-miR-200c	LEPR	1.58	0.000E+00	
hsa-miR-200c	MAP4K3	1.62	1.111E-05	involved in NSCLC metastasis[75]
hsa-miR-200c	MTUS1	2.01	0.000E+00	
hsa-miR-200c	MXD4	1.57	9.459E-06	
hsa-miR-200c	NAP1L5	1.92	0.000E+00	
hsa-miR-200c	NIPBL	2.13	0.000E+00	
hsa-miR-200c	NR2C2	1.53	3.227E-05	
hsa-miR-200c	NR3C1	1.98	0.000E+00	mediates glucocorticoids treatment in MM[76]; high levels is associated with poor prognosis in ER- breast cancer[77]
hsa-miR-200c	NUDT4	1.74	1.726E-04	
hsa-miR-200c	ORMDL3	1.88	2.054E-05	
hsa-miR-200c	OXR1	1.67	2.162E-05	
hsa-miR-200c	PCMTD1	1.76	0.000E+00	
hsa-miR-200c	PDE5A	1.59	4.483E-06	
hsa-miR-200c	PIN1	2.21	0.000E+00	
hsa-miR-200c	PPP1R10	1.76	0.000E+00	function as a proto-oncogene by sequesting PTEN[78]
hsa-miR-200c	PPP2CA	1.51	6.427E-05	

hsa-miR-200c	PRDM1	2.16	8.634E-05	mediates Ras/raf/AP-1 signaling that promotes cell migration in lung cancer[79]
hsa-miR-200c	PTPRZ1	1.54	4.016E-04	an oncogenic tyrosine phosphatase in SCLC[80]
hsa-miR-200c	RFXDC2	1.60	6.568E-06	
hsa-miR-200c	SAPS3	2.06	0.000E+00	
hsa-miR-200c	SBF1	2.27	0.000E+00	forced expression of Sbf1 induced oncogenic transformation of NIH 3T3 fibroblasts[81]
hsa-miR-200c	SMARCAD 1	1.56	4.854E-04	
hsa-miR-200c	TBC1D22B	1.63	2.693E-05	
hsa-miR-200c	TMCC1	1.87	2.945E-05	
hsa-miR-200c	TMEFF2	1.64	3.630E-06	overexpressed in prostate cancer[82]
hsa-miR-200c	TOB1	1.66	0.000E+00	
hsa-miR-200c	TRIM33	1.68	3.073E-06	
hsa-miR-200c	TSC22D2	1.54	0.000E+00	
hsa-miR-200c	TUBB3	1.57	7.221E-05	prognostic marker and predictive marker in tumors[83, 84]
hsa-miR-200c	ZFAND6	1.91	0.000E+00	
hsa-miR-200c	ZNF532	1.98	0.000E+00	amplified in glioma[85]
hsa-miR-200c, hsa-miR-135a*	DCUN1D1	1.64	0.000E+00	amplified, and oncogenic in squamous cell carcinomas[86]
hsa-miR-200c, hsa-miR-135a*	MAP2	2.26	0.000E+00	MAP2A promotes migration of oral squamous carcinoma cells[87]
hsa-miR-200c, hsa-miR-135a*	NUFIP2	1.52	0.000E+00	
hsa-miR-200c, hsa-miR-135a*	SCAMP1	2.03	0.000E+00	overexpression is associated with LN metastasis in pancreatic cancer patients[88]
hsa-miR-200c, hsa-miR-155	FBXO33	1.86	0.000E+00	
hsa-miR-200c, hsa-miR-155	GLCCI1	2.88	6.568E-06	
hsa-miR-200c, hsa-miR-155	MATR3	1.84	0.000E+00	
hsa-miR-200c, hsa-miR-155	TSGA14	1.67	0.000E+00	
hsa-miR-200c, hsa-miR-188-5p	ATXN1	1.55	1.934E-05	
hsa-miR-200c, hsa-miR-188-5p	KLF13	1.78	0.000E+00	overexpressed in oral cancer cells[89]
hsa-miR-200c, hsa-miR-188-5p	MBNL1	1.67	0.000E+00	
hsa-miR-483-5p	ABCF2	2.62	0.000E+00	higher expression associates with shorter OS in cervical cancer[90] Overexpressed in clear cell ovarian adenocarcinoma[91]
hsa-miR-630	AIG1	1.91	0.000E+00	
hsa-miR-630	ATG12	1.57	1.292E-06	
hsa-miR-630	C1orf96	1.51	5.137E-05	
hsa-miR-630	C2orf37	1.52	0.000E+00	
hsa-miR-630	CD200	1.89	0.000E+00	high expression associated with B-cell lymphoproliferative disorders[92]

hsa-miR-630	CLIC2	1.79	9.209E-05	
hsa-miR-630	CPEB4	2.05	0.000E+00	expression is heightened in glioblastomas and pancreatic ductal carcinomas, and promote tumor proliferation, invasion and vascularization[93]
hsa-miR-630	CYP51A1	1.55	4.087E-06	
hsa-miR-630	DOCK8	1.69	1.470E-05	
hsa-miR-630	GNB1	1.73	0.000E+00	higher expression in breast cancer[94]
hsa-miR-630	MEF2D	1.75	3.170E-05	
hsa-miR-630	PELI1	3.17	0.000E+00	
hsa-miR-630	PIGK	1.56	1.090E-04	
hsa-miR-630	PRKCI	1.56	3.327E-05	oncogene in lung and other cancers[95-98]
hsa-miR-630	SLC30A5	1.61	0.000E+00	
hsa-miR-630	SOCS2	1.61	6.688E-07	correlates with malignancy and exerts growth promoting effects in prostate cancer[99]
hsa-miR-630	ST3GAL6	3.29	0.000E+00	
hsa-miR-630	TDO2	1.58	0.000E+00	
hsa-miR-630	TP53RK	1.60	1.015E-05	inhibition sensitize cancers to taxanes[100]
hsa-miR-630	UPF1	2.08	0.000E+00	
hsa-miR-630	ZDHHC21	1.56	0.000E+00	
hsa-miR-630	ZNF605	1.63	0.000E+00	
hsa-miR-630	ZNF770	1.65	1.111E-05	
hsa-miR-630, hsa-miR-200c	ESRRG	2.69	0.000E+00	negatively regulates BMP2-induced osteoblast differentiation and bone formation[101]
hsa-miR-663	C7orf26	2.57	0.000E+00	
hsa-miR-663	CNN2	1.57	1.272E-05	
hsa-miR-663	FKBP8	2.01	7.839E-06	protects BCL-2 from caspase-dependent degradation[102]

Supplementary Table S4.

1. Gumulec J, Masarik M, Krizkova S, Hlavna M, Babula P, Hrabec R, Rovny A, Masarikova M, Sochor J, Adam V, Eckschlager T and Kizek R. Evaluation of alpha-methylacyl-CoA racemase, metallothionein and prostate specific antigen as prostate cancer prognostic markers. Neoplasma. 2012; 59(2):191-201.

2. Hirakawa M, Takimoto R, Tamura F, Yoshida M, Ono M, Murase K, Sato Y, Osuga T, Sato T, Iyama S, Miyanishi K, Takada K, Hayashi T, Kobune M and Kato J. Fucosylated TGF-beta receptors transduces a signal for epithelial-mesenchymal transition in colorectal cancer cells. Br J Cancer. 2013.

3. Powell AA, Talasaz AH, Zhang H, Coram MA, Reddy A, Deng G, Telli ML, Advani RH, Carlson RW, Mollick JA, Sheth S, Kurian AW, Ford JM, Stockdale FE, Quake SR, Pease RF, et al. Single cell profiling of circulating tumor cells: transcriptional heterogeneity and diversity from breast cancer cell lines. PLoS One. 2012; 7(5):e33788.

4. Rodriguez-Pinto D, Sparkowski J, Keough MP, Phoenix KN, Vumbaca F, Han DK, Gundelfinger ED, Beesley P and Claffey KP. Identification of novel tumor antigens with patient-derived immune-selected antibodies. Cancer immunology, immunotherapy : CII. 2009; 58(2):221-234.

5. Oike T, Ogiwara H, Tominaga Y, Ito K, Ando O, Tsuta K, Mizukami T, Shimada Y, Isomura H, Komachi M, Furuta K, Watanabe S, Nakano T, Yokota J and Kohno T. A synthetic lethality-based strategy to treat cancers harboring a genetic deficiency in the chromatin remodeling factor BRG1. Cancer Res. 2013; 73(17):5508-5518.

6. Bhattacharya C, Wang X and Becker D. The DEAD/DEAH box helicase, DDX11, is essential for the survival of advanced melanomas. Molecular cancer. 2012; 11:82.

7. Du JW, Xu KY, Fang LY and Qi XL. Clinical significance of Mena and Her-2 expression in breast cancer. European journal of gynaecological oncology. 2012; 33(5):455-458.

8. Roussos ET, Goswami S, Balsamo M, Wang Y, Stobezki R, Adler E, Robinson BD, Jones JG, Gertler FB, Condeelis JS and Oktay MH. Mena invasive (Mena(INV)) and Mena11a isoforms play distinct roles in breast cancer cell cohesion and association with TMEM. Clinical & experimental metastasis. 2011; 28(6):515-527.

9. Li Y, Zhao L, Sun H, Yu J, Li N, Liang J, Wang Y, He M, Bai X, Yu Z, Zheng Z, Mi X, Wang E and Wei M. Gene silencing of FANCF potentiates the sensitivity to mitoxantrone through activation of JNK and p38 signal pathways in breast cancer cells. PLoS One. 2012; 7(8):e44254.

10. Ro TB, Holien T, Fagerli UM, Hov H, Misund K, Waage A, Sundan A, Holt RU and Borset M. HGF and IGF-1 synergize with SDF-1alpha in promoting migration of myeloma cells by cooperative activation of p21-activated kinase. Exp Hematol. 2013; 41(7):646-655.

11. Kristensen IB, Pedersen L, Ro TB, Christensen JH, Lyng MB, Rasmussen LM, Ditzel HJ, Borset M and Abildgaard N. Decorin is down-regulated in multiple myeloma and MGUS bone marrow plasma and inhibits HGF-induced myeloma plasma cell viability and migration. Eur J Haematol. 2013; 91(3):196-200.

12. Choi S, Thapa N, Hedman AC, Li Z, Sacks DB and Anderson RA. IQGAP1 is a novel phosphatidylinositol 4,5 bisphosphate effector in regulation of directional cell migration. The EMBO journal. 2013; 32(19):2617-2630.

13. Alemayehu M, Dragan M, Pape C, Siddiqui I, Sacks DB, Di Guglielmo GM, Babwah AV and Bhattacharya M. beta-Arrestin2 regulates lysophosphatidic acid-induced human breast tumor cell migration and invasion via Rap1 and IQGAP1. PLoS One. 2013; 8(2):e56174.

14. Wang XX, Li XZ, Zhai LQ, Liu ZR, Chen XJ and Pei Y. Overexpression of IQGAP1 in human

pancreatic cancer. Hepatobiliary & pancreatic diseases international : HBPD INT. 2013; 12(5):540-545.

15. Jameson KL, Mazur PK, Zehnder AM, Zhang J, Zarnegar B, Sage J and Khavari PA. IQGAP1 scaffold-kinase interaction blockade selectively targets RAS-MAP kinase-driven tumors. Nat Med. 2013; 19(5):626-630.

16. Frau M, Feo F and Pascale RM. Pleiotropic effects of methionine adenosyltransferases deregulation as determinants of liver cancer progression and prognosis. Journal of hepatology. 2013; 59(4):830-841.

17. Tan Z, Li M, Wu W, Zhang L, Ding Q, Wu X, Mu J and Liu Y. NLK is a key regulator of proliferation and migration in gallbladder carcinoma cells. Molecular and cellular biochemistry. 2012; 369(1-2):27-33.

18. Jung KH, Kim JK, Noh JH, Eun JW, Bae HJ, Xie HJ, Ahn YM, Park WS, Lee JY and Nam SW. Targeted disruption of Nemo-like kinase inhibits tumor cell growth by simultaneous suppression of cyclin D1 and CDK2 in human hepatocellular carcinoma. Journal of cellular biochemistry. 2010; 110(3):687-696.

19. Li H, Cai Q, Wu H, Vathipadiekal V, Dobbin ZC, Li T, Hua X, Landen CN, Birrer MJ, Sanchez-Beato M and Zhang R. SUZ12 promotes human epithelial ovarian cancer by suppressing apoptosis via silencing HRK. Molecular cancer research : MCR. 2012; 10(11):1462-1472.

20. Mann KM, Ward JM, Yew CC, Kovochich A, Dawson DW, Black MA, Brett BT, Sheetz TE, Dupuy AJ, Australian Pancreatic Cancer Genome I, Chang DK, Biankin AV, Waddell N, Kassahn KS, Grimmond SM, Rust AG, et al. Sleeping Beauty mutagenesis reveals cooperating mutations and pathways in pancreatic adenocarcinoma. Proc Natl Acad Sci U S A. 2012; 109(16):5934-5941.

21. Bergsagel PL, Kuehl WM, Zhan F, Sawyer J, Barlogie B and Shaughnessy J, Jr. Cyclin D dysregulation: an early and unifying pathogenic event in multiple myeloma. Blood. 2005; 106(1):296-303.

22. Musgrove EA, Caldon CE, Barraclough J, Stone A and Sutherland RL. Cyclin D as a therapeutic target in cancer. Nat Rev Cancer. 2011; 11(8):558-572.

23. Yoshida H, Tomiyama Y, Ishikawa J, Oritani K, Matsumura I, Shiraga M, Yokota T, Okajima Y, Ogawa M, Miyagawa J, Nishiura T and Matsuzawa Y. Integrin-associated protein/CD47 regulates motile activity in human B-cell lines through CDC42. Blood. 2000; 96(1):234-241.

24. Uluckan O, Becker SN, Deng H, Zou W, Prior JL, Piwnica-Worms D, Frazier WA and Weilbaecher KN. CD47 regulates bone mass and tumor metastasis to bone. Cancer Res. 2009; 69(7):3196-3204.

25. Kim D, Wang J, Willingham SB, Martin R, Wernig G and Weissman IL. Anti-CD47 antibodies promote phagocytosis and inhibit the growth of human myeloma cells. Leukemia. 2012; 26(12):2538-2545.

26. Wang Z, Liu H, Liu B, Ma W, Xue X, Chen J and Zhou Q. Gene expression levels of CSNK1A1 and AAC-11, but not NME1, in tumor tissues as prognostic factors in NSCLC patients. Medical science monitor : international medical journal of experimental and clinical research. 2010; 16(8):CR357-364.

27. Tominaga E, Tsuda H, Arao T, Nishimura S, Takano M, Kataoka F, Nomura H, Hirasawa A, Aoki D and Nishio K. Amplification of GNAS may be an independent, qualitative, and reproducible biomarker to predict progression-free survival in epithelial ovarian cancer. Gynecol Oncol. 2010; 118(2):160-166.

28. Zhao LY, Zhang J, Guo B, Yang J, Han J, Zhao XG, Wang XF, Liu LY, Li ZF, Song TS and Huang C. MECP2 promotes cell proliferation by activating ERK1/2 and inhibiting p38 activity in human

hepatocellular carcinoma HEPG2 cells. Cellular and molecular biology. 2013; Suppl 59:OL1876-1881.

29. Shen Z, Zeng Y, Guo J, Wu Y, Jiang X, Ding R, Wu C, Li R, Luo B, Zeng C, Jiang H and Jie W. Over-expression of the special AT rich sequence binding protein 1 (SATB1) promotes the progression of nasopharyngeal carcinoma: association with EBV LMP-1 expression. Journal of translational medicine. 2013; 11(1):217.

30. Shukla S, Sharma H, Abbas A, MacLennan GT, Fu P, Danielpour D and Gupta S. Upregulation of SATB1 is associated with prostate cancer aggressiveness and disease progression. PLoS One. 2013; 8(1):e53527.

31. Bai J, Mei P, Zhang C, Chen F, Li C, Pan Z, Liu H and Zheng J. BRG1 is a prognostic marker and potential therapeutic target in human breast cancer. PLoS One. 2013; 8(3):e59772.

32. Magnussen GI, Holm R, Emilsen E, Rosnes AK, Slipicevic A and Florenes VA. High expression of Wee1 is associated with poor disease-free survival in malignant melanoma: potential for targeted therapy. PLoS One. 2012; 7(6):e38254.

33. Ren M, Li X and Cowell JK. Genetic fingerprinting of the development and progression of T-cell lymphoma in a murine model of atypical myeloproliferative disorder initiated by the ZNF198-fibroblast growth factor receptor-1 chimeric tyrosine kinase. Blood. 2009; 114(8):1576-1584.

34. Wang M, Zhang D, Wang R, Rui Y, Zhou J, Wang R, Zhou B, Huang X, Yang L, Li Y, Hu J, Zhou Z and Sun X. A-kinase anchoring proteins 10 expression in relation to 2073A/G polymorphism and tumor progression in patients with colorectal cancer. Pathology oncology research : POR. 2013; 19(3):521-527.

35. Mani M, Carrasco DE, Zhang Y, Takada K, Gatt ME, Dutta-Simmons J, Ikeda H, Diaz-Griffero F, Pena-Cruz V, Bertagnolli M, Myeroff LL, Markowitz SD, Anderson KC and Carrasco DR. BCL9 promotes tumor progression by conferring enhanced proliferative, metastatic, and angiogenic properties to cancer cells. Cancer Res. 2009; 69(19):7577-7586.

36. Takada K, Zhu D, Bird GH, Sukhdeo K, Zhao JJ, Mani M, Lemieux M, Carrasco DE, Ryan J, Horst D, Fulciniti M, Munshi NC, Xu W, Kung AL, Shivdasani RA, Walensky LD, et al. Targeted disruption of the BCL9/beta-catenin complex inhibits oncogenic Wnt signaling. Science translational medicine. 2012; 4(148):148ra117.

37. Jiang T, Tang HM, Wu ZH, Chen J, Lu S, Zhou CZ, Yan DW and Peng ZH. Cullin 4B is a novel prognostic marker that correlates with colon cancer progression and pathogenesis. Medical oncology. 2013; 30(2):534.

38. Hu H, Yang Y, Ji Q, Zhao W, Jiang B, Liu R, Yuan J, Liu Q, Li X, Zou Y, Shao C, Shang Y, Wang Y and Gong Y. CRL4B catalyzes H2AK119 monoubiquitination and coordinates with PRC2 to promote tumorigenesis. Cancer Cell. 2012; 22(6):781-795.

39. Cooper LA, Gutman DA, Long Q, Johnson BA, Cholleti SR, Kurc T, Saltz JH, Brat DJ and Moreno CS. The proneural molecular signature is enriched in oligodendrogliomas and predicts improved survival among diffuse gliomas. PLoS One. 2010; 5(9):e12548.

40. Lukashova-v Zangen I, Kneitz S, Monoranu CM, Rutkowski S, Hinkes B, Vince GH, Huang B and Roggendorf W. Ependymoma gene expression profiles associated with histological subtype, proliferation, and patient survival. Acta neuropathologica. 2007; 113(3):325-337.

41. Moreno DA, Scrideli CA, Cortez MA, de Paula Queiroz R, Valera ET, da Silva Silveira V, Yunes JA, Brandalise SR and Tone LG. Differential expression of HDAC3, HDAC7 and HDAC9 is associated with prognosis and survival in childhood acute lymphoblastic leukaemia. Br J Haematol. 2010; 150(6):665-673.

42. Hu Q, Lu YY, Noh H, Hong S, Dong Z, Ding HF, Su SB and Huang S. Interleukin enhancer-

binding factor 3 promotes breast tumor progression by regulating sustained urokinase-type plasminogen activator expression. Oncogene. 2013; 32(34):3933-3943.

43. Kasper G, Vogel A, Klaman I, Grone J, Petersen I, Weber B, Castanos-Velez E, Staub E and Mennerich D. The human LAPTM4b transcript is upregulated in various types of solid tumours and seems to play a dual functional role during tumour progression. Cancer letters. 2005; 224(1):93-103.

44. Yang H, Xiong F, Qi R, Liu Z, Lin M, Rui J, Su J and Zhou R. LAPTM4B-35 is a novel prognostic factor of hepatocellular carcinoma. J Surg Oncol. 2010; 101(5):363-369.

45. Fan Y, Ge N, Wang X, Sun W, Mao R, Bu W, Creighton CJ, Zheng P, Vasudevan S, An L, Yang J, Zhao YJ, Zhang H, Li XN, Rao PH, Leung E, et al. Amplification and overexpression of MAP3K3 gene in human breast cancer promotes formation and survival of breast cancer cells. J Pathol. 2013.

46. Agatheeswaran S, Singh S, Biswas S, Biswas G, Chandra Pattnayak N and Chakraborty S. BCR-ABL mediated repression of miR-223 results in the activation of MEF2C and PTBP2 in chronic myeloid leukemia. Leukemia. 2013; 27(7):1578-1580.

47. Homminga I, Pieters R, Langerak AW, de Rooi JJ, Stubbs A, Verstegen M, Vuerhard M, Buijs-Gladdines J, Kooi C, Klous P, van Vlierberghe P, Ferrando AA, Cayuela JM, Verhaaf B, Beverloo HB, Horstmann M, et al. Integrated transcript and genome analyses reveal NKX2-1 and MEF2C as potential oncogenes in T cell acute lymphoblastic leukemia. Cancer Cell. 2011; 19(4):484-497.

48. Schwieger M, Schuler A, Forster M, Engelmann A, Arnold MA, Delwel R, Valk PJ, Lohler J, Slany RK, Olson EN and Stocking C. Homing and invasiveness of MLL/ENL leukemic cells is regulated by MEF2C. Blood. 2009; 114(12):2476-2488.

49. O'Neal J, Gao F, Hassan A, Monahan R, Barrios S, Kilimann MW, Lee I, Chng WJ, Vij R and Tomasson MH. Neurobeachin (NBEA) is a target of recurrent interstitial deletions at 13q13 in patients with MGUS and multiple myeloma. Exp Hematol. 2009; 37(2):234-244.

50. Kim TJ, Choi JJ, Kim WY, Choi CH, Lee JW, Bae DS, Son DS, Kim J, Park BK, Ahn G, Cho EY and Kim BG. Gene expression profiling for the prediction of lymph node metastasis in patients with cervical cancer. Cancer science. 2008; 99(1):31-38.

51. Koensgen D, Mustea A, Klaman I, Sun P, Zafrakas M, Lichtenegger W, Denkert C, Dahl E and Sehouli J. Expression analysis and RNA localization of PAI-RBP1 (SERBP1) in epithelial ovarian cancer: association with tumor progression. Gynecol Oncol. 2007; 107(2):266-273.

52. Garzon-Muvdi T, Schiapparelli P, ap Rhys C, Guerrero-Cazares H, Smith C, Kim DH, Kone L, Farber H, Lee DY, An SS, Levchenko A and Quinones-Hinojosa A. Regulation of brain tumor dispersal by NKCC1 through a novel role in focal adhesion regulation. PLoS biology. 2012; 10(5):e1001320.

53. Johnson MD and O'Connell M. Na-K-2Cl cotransporter and aquaporin 1 in arachnoid granulations, meningiomas, and meningiomas invading dura. Human pathology. 2013; 44(6):1118-1124.

54. Njiaju UO, Gamazon ER, Gorsic LK, Delaney SM, Wheeler HE, Im HK and Dolan ME. Whole-genome studies identify solute carrier transporters in cellular susceptibility to paclitaxel. Pharmacogenet Genomics. 2012; 22(7):498-507.

55. Peschiaroli A, Skaar JR, Pagano M and Melino G. The ubiquitin-specific protease USP47 is a novel beta-TRCP interactor regulating cell survival. Oncogene. 2010; 29(9):1384-1393.

56. Brito JL, Walker B, Jenner M, Dickens NJ, Brown NJ, Ross FM, Avramidou A, Irving JA, Gonzalez D, Davies FE and Morgan GJ. MMSET deregulation affects cell cycle progression and adhesion regulons in t(4;14) myeloma plasma cells. Haematologica. 2009; 94(1):78-86.

57. Min DJ, Ezponda T, Kim MK, Will CM, Martinez-Garcia E, Popovic R, Basrur V, Elenitoba-

Johnson KS and Licht JD. MMSET stimulates myeloma cell growth through microRNA-mediated modulation of c-MYC. Leukemia. 2013; 27(3):686-694.

58. Agarwal NK, Chen CH, Cho H, Boulbes DR, Spooner E and Sarbassov DD. Rictor regulates cell migration by suppressing RhoGDI2. Oncogene. 2013; 32(20):2521-2526.

59. Im-Aram A, Farrand L, Bae SM, Song G, Song YS, Han JY and Tsang BK. The mTORC2 Component Rictor Contributes to Cisplatin Resistance in Human Ovarian Cancer Cells. PLoS One. 2013; 8(9):e75455.

60. Howe EN, Cochrane DR and Richer JK. Targets of miR-200c mediate suppression of cell motility and anoikis resistance. Breast Cancer Res. 2011; 13(2):R45.

61. Ren GF, Tang L, Yang AQ, Jiang WW and Huang YM. Prognostic impact of NDRG2 and NDRG3 in prostate cancer patients undergoing radical prostatectomy. Histology and histopathology. 2013.

62. Wang W, Li Y, Li Y, Hong A, Wang J, Lin B and Li R. NDRG3 is an androgen regulated and prostate enriched gene that promotes in vitro and in vivo prostate cancer cell growth. Int J Cancer. 2009; 124(3):521-530.

63. Almahariq M, Tsalkova T, Mei FC, Chen H, Zhou J, Sastry SK, Schwede F and Cheng X. A novel EPAC-specific inhibitor suppresses pancreatic cancer cell migration and invasion. Molecular pharmacology. 2013; 83(1):122-128.

64. Baljinnyam E, De Lorenzo MS, Xie LH, Iwatsubo M, Chen S, Goydos JS, Nowycky MC and Iwatsubo K. Exchange protein directly activated by cyclic AMP increases melanoma cell migration by a Ca2+-dependent mechanism. Cancer Res. 2010; 70(13):5607-5617.

65. Tachibana M, Kiyokawa E, Hara S, Iemura S, Natsume T, Manabe T and Matsuda M. Ankyrin repeat domain 28 (ANKRD28), a novel binding partner of DOCK180, promotes cell migration by regulating focal adhesion formation. Experimental cell research. 2009; 315(5):863-876.

66. Yamashita T, Okamura T, Nagano K, Imai S, Abe Y, Nabeshi H, Yoshikawa T, Yoshioka Y, Kamada H, Tsutsumi Y and Tsunoda S. Rho GDP-dissociation inhibitor alpha is associated with cancer metastasis in colon and prostate cancer. Die Pharmazie. 2012; 67(3):253-255.

67. Rong F, Li W, Chen K, Li DM, Duan WM, Feng YZ, Li F, Zhou XW, Fan SJ, Liu Y and Tao M. Knockdown of RhoGDIalpha induces apoptosis and increases lung cancer cell chemosensitivity to paclitaxel. Neoplasma. 2012; 59(5):541-550.

68. Vimala K, Sundarraj S, Sujitha MV and Kannan S. Curtailing overexpression of E2F3 in breast cancer using siRNA (E2F3)-based gene silencing. Archives of medical research. 2012; 43(6):415-422.

69. Feng B, Wang R, Song HZ and Chen LB. MicroRNA-200b reverses chemoresistance of docetaxel-resistant human lung adenocarcinoma cells by targeting E2F3. Cancer. 2012; 118(13):3365-3376.

70. Bonetti P, Testoni M, Scandurra M, Ponzoni M, Piva R, Mensah AA, Rinaldi A, Kwee I, Tibiletti MG, Iqbal J, Greiner TC, Chan WC, Gaidano G, Piris MA, Cavalli F, Zucca E, et al. Deregulation of ETS1 and FLI1 contributes to the pathogenesis of diffuse large B-cell lymphoma. Blood. 2013; 122(13):2233-2241.

71. Cai C, Rajaram M, Zhou X, Liu Q, Marchica J, Li J and Powers RS. Activation of multiple cancer pathways and tumor maintenance function of the 3q amplified oncogene FNDC3B. Cell Cycle. 2012; 11(9):1773-1781.

72. Yang ZF, Zhang H, Ma L, Peng C, Chen Y, Wang J, Green MR, Li S and Rosmarin AG. GABP transcription factor is required for development of chronic myelogenous leukemia via its control of PRKD2. Proc Natl Acad Sci U S A. 2013; 110(6):2312-2317.

73. Yu S, Cui K, Jothi R, Zhao DM, Jing X, Zhao K and Xue HH. GABP controls a critical transcription regulatory module that is essential for maintenance and differentiation of hematopoietic stem/progenitor cells. Blood. 2011; 117(7):2166-2178.

74. Ke HL, Ke RH, Li B, Wang XH, Wang YN and Wang XQ. Association between laminin gamma1 expression and meningioma grade, recurrence, and progression-free survival. Acta neurochirurgica. 2013; 155(1):165-171.

75. Zhao B, Han H, Chen J, Zhang Z, Li S, Fang F, Zheng Q, Ma Y, Zhang J, Wu N and Yang Y. MicroRNA let-7c inhibits migration and invasion of human non-small cell lung cancer by targeting ITGB3 and MAP4K3. Cancer letters. 2014; 342(1):43-51.

76. Heuck CJ, Szymonifka J, Hansen E, Shaughnessy JD, Jr., Usmani SZ, van Rhee F, Anaissie E, Nair B, Waheed S, Alsayed Y, Petty N, Bailey C, Epstein J, Hoering A, Crowley J and Barlogie B. Thalidomide in total therapy 2 overcomes inferior prognosis of myeloma with low expression of the glucocorticoid receptor gene NR3C1. Clin Cancer Res. 2012; 18(19):5499-5506.

77. Pan D, Kocherginsky M and Conzen SD. Activation of the glucocorticoid receptor is associated with poor prognosis in estrogen receptor-negative breast cancer. Cancer Res. 2011; 71(20):6360-6370.

78. Kavela S, Shinde SR, Ratheesh R, Viswakalyan K, Bashyam MD, Gowrishankar S, Vamsy M, Pattnaik S, Rao S, Sastry RA, Srinivasulu M, Chen J and Maddika S. PNUTS functions as a proto-oncogene by sequestering PTEN. Cancer Res. 2013; 73(1):205-214.

79. Yu Z, Sato S, Trackman PC, Kirsch KH and Sonenshein GE. Blimp1 activation by AP-1 in human lung cancer cells promotes a migratory phenotype and is inhibited by the lysyl oxidase propeptide. PLoS One. 2012; 7(3):e33287.

80. Makinoshima H, Ishii G, Kojima M, Fujii S, Higuchi Y, Kuwata T and Ochiai A. PTPRZ1 regulates calmodulin phosphorylation and tumor progression in small-cell lung carcinoma. BMC Cancer. 2012; 12:537.

81. Cui X, De Vivo I, Slany R, Miyamoto A, Firestein R and Cleary ML. Association of SET domain and myotubularin-related proteins modulates growth control. Nat Genet. 1998; 18(4):331-337.

82. Afar DE, Bhaskar V, Ibsen E, Breinberg D, Henshall SM, Kench JG, Drobnjak M, Powers R, Wong M, Evangelista F, O'Hara C, Powers D, DuBridge RB, Caras I, Winter R, Anderson T, et al. Preclinical validation of anti-TMEFF2-auristatin E-conjugated antibodies in the treatment of prostate cancer. Mol Cancer Ther. 2004; 3(8):921-932.

83. Seve P and Dumontet C. Is class III beta-tubulin a predictive factor in patients receiving tubulin-binding agents? Lancet Oncol. 2008; 9(2):168-175.

84. Hwang JE, Hong JY, Kim K, Kim SH, Choi WY, Kim MJ, Jung SH, Shim HJ, Bae WK, Hwang EC, Lee KH, Lee JH, Cho SH and Chung IJ. Class III beta-tubulin is a predictive marker for taxane-based chemotherapy in recurrent and metastatic gastric cancer. BMC Cancer. 2013; 13:431.

85. Cowell JK, Lo KC, Luce J and Hawthorn L. Interpreting aCGH-defined karyotypic changes in gliomas using copy number status, loss of heterozygosity and allelic ratios. Experimental and molecular pathology. 2010; 88(1):82-89.

86. Sarkaria I, P Oc, Talbot SG, Reddy PG, Ngai I, Maghami E, Patel KN, Lee B, Yonekawa Y, Dudas M, Kaufman A, Ryan R, Ghossein R, Rao PH, Stoffel A, Ramanathan Y, et al. Squamous cell carcinoma related oncogene/DCUN1D1 is highly conserved and activated by amplification in squamous cell carcinomas. Cancer Res. 2006; 66(19):9437-9444.

87. Liu SY, Chen YT, Tseng MY, Hung CC, Chiang WF, Chen HR, Shieh TY, Chen CH, Jou YS and Chen JY. Involvement of microtubule-associated protein 2 (MAP2) in oral cancer cell motility: a novel

biological function of MAP2 in non-neuronal cells. Biochem Biophys Res Commun. 2008; 366(2):520-525.

88. Yang S, Lee KT, Lee JY, Lee JK, Lee KH and Rhee JC. Inhibition of SCAMP1 suppresses cell migration and invasion in human pancreatic and gallbladder cancer cells. Tumour biology : the journal of the International Society for Oncodevelopmental Biology and Medicine. 2013; 34(5):2731-2739.

89. Henson BJ and Gollin SM. Overexpression of KLF13 and FGFR3 in oral cancer cells. Cytogenetic and genome research. 2010; 128(4):192-198.

90. Nishimura S, Tsuda H, Miyagi Y, Hirasawa A, Suzuki A, Kataoka F, Nomura H, Chiyoda T, Banno K, Fujii T, Susumu N and Aoki D. Can ABCF2 protein expression predict the prognosis of uterine cancer? Br J Cancer. 2008; 99(10):1651-1655.

91. Tsuda H, Ito YM, Ohashi Y, Wong KK, Hashiguchi Y, Welch WR, Berkowitz RS, Birrer MJ and Mok SC. Identification of overexpression and amplification of ABCF2 in clear cell ovarian adenocarcinomas by cDNA microarray analyses. Clin Cancer Res. 2005; 11(19 Pt 1):6880-6888.

92. El Desoukey NA, Afify RA, Amin DG and Mohammed RF. CD200 expression in B-cell chronic lymphoproliferative disorders. Journal of investigative medicine : the official publication of the American Federation for Clinical Research. 2012; 60(1):56-61.

93. Ortiz-Zapater E, Pineda D, Martinez-Bosch N, Fernandez-Miranda G, Iglesias M, Alameda F, Moreno M, Eliscovich C, Eyras E, Real FX, Mendez R and Navarro P. Key contribution of CPEB4-mediated translational control to cancer progression. Nat Med. 2012; 18(1):83-90.

94. Wazir U, Jiang WG, Sharma AK and Mokbel K. Guanine nucleotide binding protein beta 1: a novel transduction protein with a possible role in human breast cancer. Cancer genomics & proteomics. 2013; 10(2):69-73.

95. Wang Y, Hill KS and Fields AP. Protein Kinase Ciota maintains a tumor-initiating cell phenotype that is required for ovarian tumorigenesis. Molecular cancer research : MCR. 2013.

96. Atwood SX, Li M, Lee A, Tang JY and Oro AE. GLI activation by atypical protein kinase C iota/lambda regulates the growth of basal cell carcinomas. Nature. 2013; 494(7438):484-488.

97. Kikuchi K, Soundararajan A, Zarzabal LA, Weems CR, Nelon LD, Hampton ST, Michalek JE, Rubin BP, Fields AP and Keller C. Protein kinase C iota as a therapeutic target in alveolar rhabdomyosarcoma. Oncogene. 2013; 32(3):286-295.

98. Yang YL, Chu JY, Luo ML, Wu YP, Zhang Y, Feng YB, Shi ZZ, Xu X, Han YL, Cai Y, Dong JT, Zhan QM, Wu M and Wang MR. Amplification of PRKCI, located in 3q26, is associated with lymph node metastasis in esophageal squamous cell carcinoma. Genes Chromosomes Cancer. 2008; 47(2):127-136.

99. Hoefer J, Kern J, Ofer P, Eder IE, Schafer G, Dietrich D, Kristiansen G, Geley S, Rainer J, Gunsilius E, Klocker H, Culig Z and Puhr M. SOCS2 correlates with malignancy and exerts growth promoting effects in prostate cancer. Endocrine-related cancer. 2013.

100. Peterson D, Lee J, Lei XC, Forrest WF, Davis DP, Jackson PK and Belmont LD. A chemosensitization screen identifies TP53RK, a kinase that restrains apoptosis after mitotic stress. Cancer Res. 2010; 70(15):6325-6335.

101. Jeong BC, Lee YS, Park YY, Bae IH, Kim DK, Koo SH, Choi HR, Kim SH, Franceschi RT, Koh JT and Choi HS. The orphan nuclear receptor estrogen receptor-related receptor gamma negatively regulates BMP2-induced osteoblast differentiation and bone formation. J Biol Chem. 2009; 284(21):14211-14218.

102. Choi BH, Feng L and Yoon HS. FKBP38 protects Bcl-2 from caspase-dependent degradation. J Biol Chem. 2010; 285(13):9770-9779.

Supplementary Table S5. GSEA analysis, using the 215 predicted miRNA targets that are also upregulated in MM patients

NAME	SIZE	NES	NOM p-val	FDR q-val
ZHENG_BOUND_BY_FOXP3[1]	15	2.07	0.003	0.249
HELLER_HDAC_TARGETS_DN[2]	10	1.91	0.004	0.382
SMID_BREAST_CANCER_BASAL_DN[3]	10	1.89	0.008	0.303
**HELLER_HDAC_TARGETS_SILENCED_BY_METHYLATION_DN[2]**	**13**	**1.88**	**0.018**	**0.243**

References

1. Zheng Y, Josefowicz SZ, Kas A, Chu TT, Gavin MA and Rudensky AY. Genome-wide analysis of Foxp3 target genes in developing and mature regulatory T cells. Nature. 2007; 445(7130):936-940.
2. Heller G, Schmidt WM, Ziegler B, Holzer S, Mullauer L, Bilban M, Zielinski CC, Drach J and Zochbauer-Muller S. Genome-wide transcriptional response to 5-aza-2'-deoxycytidine and trichostatin a in multiple myeloma cells. Cancer Res. 2008; 68(1):44-54.
3. Smid M, Wang Y, Zhang Y, Sieuwerts AM, Yu J, Klijn JG, Foekens JA and Martens JW. Subtypes of breast cancer show preferential site of relapse. Cancer Res. 2008; 68(9):3108-3114.

Supplementary Table S6. KEGG Pathway Analysis of the predicted targets of leading candidate TS-miRNAs

Database Name	Pathway Name	p-value	Unique Pathway-id
KEGG	Small cell lung cancer	1.049E-03	1:05222
KEGG	Melanoma	3.762E-03	1:05218
KEGG	TGF-beta signaling pathway	7.719E-03	1:04350
KEGG	Prostate cancer	8.680E-03	1:05215
KEGG	Pathways in cancer	9.876E-03	1:05200
KEGG	Wnt signaling pathway	1.190E-02	1:04310
KEGG	Glioma	2.043E-02	1:05214
KEGG	p53 signaling pathway	2.389E-02	1:04115
KEGG	Pancreatic cancer	2.667E-02	1:05212
KEGG	Chronic myeloid leukemia	2.962E-02	1:05220
KEGG	Adherens junction	3.273E-02	1:04520
KEGG	Ubiquitin mediated proteolysis	3.623E-02	1:04120
KEGG	Colorectal cancer	3.945E-02	1:05210

Supplementary Table S7. 33 genes independently associated with survival in UAMS dataset are predicted to be targeted by leading candidate TS-miRNAs.

miRNA ID	Predicted Target	No.of leading miRNAs predicted to target it	Fold increase in MM	Adjusted P-value
hsa-miR-125a-3p	CYCS	1	1.92	0.000E+00
hsa-miR-200c	DCUN1D1	2	1.64	0.000E+00
hsa-miR-135a*	DCUN1D1	2	1.64	0.000E+00
hsa-miR-198	FAM55C	1	1.61	2.594E-05
hsa-miR-135a*	VGLL4	1	1.55	0.000E+00
hsa-miR-483-5p	ABCF2	1	2.62	0.000E+00
hsa-miR-135a*	ALS2	1	1.71	0.000E+00
hsa-miR-198	ARHGAP19	1	1.8	0.000E+00
hsa-miR-630	C1orf96	1	1.51	5.137E-05
hsa-miR-135a*	DDX11	1	1.8	0.000E+00
hsa-miR-125a-3p	FAHD1	1	1.55	3.073E-06
hsa-miR-198	FANCL	1	1.53	4.087E-06
hsa-miR-125a-3p	GMCL1	1	1.51	6.568E-06
hsa-miR-135a*	NUP160	1	1.76	0.000E+00
hsa-miR-188-5p	RBM8A	1	1.97	0.000E+00
hsa-miR-200c	RFXDC2	1	1.6	6.568E-06
hsa-miR-200c	TUBB3	1	1.57	7.221E-05
hsa-miR-198	VBP1	1	1.55	6.688E-07
hsa-miR-155	WEE1	1	1.5	0.000E+00
hsa-miR-188-5p	WHSC1	1	2.32	0.000E+00
hsa-miR-200c	NR3C1	1	1.98	0.000E+00
hsa-miR-125a-3p	ZFP3	1	1.74	3.227E-05
hsa-miR-188-5p	ZNF197	2	1.61	6.568E-06
hsa-miR-200c	BAZ2B	1	1.77	0.000E+00
hsa-miR-155	CCND1	1	2.8	0.000E+00
hsa-miR-200c	LEPR	1	1.58	0.000E+00
hsa-miR-200c	MAP4K3	1	1.62	1.111E-05
hsa-miR-200c	MTUS1	1	2.01	0.000E+00
hsa-miR-200c	NAP1L5	1	1.92	0.000E+00
hsa-miR-135a*	NLK	1	1.69	1.292E-06
hsa-miR-200c	ORMDL3	1	1.88	2.054E-05
hsa-miR-200c	PCMTD1	1	1.76	0.000E+00
hsa-miR-125a-3p	SMARCA2	1	2.01	0.000E+00
hsa-miR-155	ZMYM2	1	1.55	3.733E-04

Supplementary Table S8. Fold change after 5'aza treatment for known TS-miRNAs silenced by DNA methylation

	H929	MM1S	OPM2	8226
miR-194	15.97	n.d	2.50	1.81
miR-192	1.42	3.16	1.55	1.14
miR-215	4.66	n.d	1.75	1.42
miR-29b	1.90	1.68	1.66	1.06
miR-34a	1.05	0.90	6.63	0.48
miR-34b	n.d	n.d	n.d	n.d
miR-34b*	0.86	1.70	n.d	n.d
miR-34c	n.d	n.d	n.d	n.d
miR-203	n.d	n.d	n.d	n.d

n.d = not detected

Supplementary Table S9. MSP primer sequences and annealing temperatures used in the PCR reactions, and product size.

Name of mir	Sequence	Annealing Temperature (°C)	Product Size (bp)
mir-155M-F	GTCGAGTTCGGGTTTAGC	54	134
mir-155M-R	GCGAAACTAAAATCGACGTAC	54	134
mir-155U-F	GTTGAGTTTGGGTTTAGT	54	134
mir-155U-R	ACAAAACTAAAATCAACATAC	54	134
mir-198M-F	CGTTTTACGTTTAGGGGGTC	54	142
mir-198M-R	TACTACAACGACCCCCGC	54	142
mir-198U-F	TTTTGTTTTATGTTTAGGGGGTT	54	142
mir-198U-R	TCTACTACAACAACCCCCACA	54	142
mir-200M-F	GTTTTTCGTTTTGAGTTGAGAGC	60	125
mir-200M-R	CTAAATCCACCAAATAACAAATCG	60	125
mir-200U-F	TTTTTGTTTTGAGTTGAGAGTGT	60	125
mir-200U-R	CTAAATCCACCAAATAACAAATCAC	60	125
mir-663M-F	CGTTCGTTTTTTTGTCGAGTC	60	161
mir-663M-R	ACCACATCGCTCGTAATTCTC	60	161
mir-663U-F	TGTTTGTTTTTTTGTTGAGTT	60	161
mir-663U-R	ACCACATCACTCATAATTCTC	60	161
